# A Simplified Three‐Tailed N‐Alkyl Phosphoramidate Lipid Platform Enables Inguinal Adipose‐Accumulated mRNA Delivery for Anti‐Obesity Therapy

**DOI:** 10.1002/advs.202517672

**Published:** 2025-12-22

**Authors:** Bin Ma, Yunxuan Liu, Huijuan Zhang, Yian Fang, Yizhe Xue, Junsheng Xue, Ziqiong Jiang, Tianyan Zhou, Yanyun Hao, Fei Xie, Lei Miao

**Affiliations:** ^1^ State Key Laboratory of Natural and Biomimetic Drugs School of Pharmaceutical Sciences Peking University Beijing P. R. China; ^2^ Beijing Key Laboratory of Molecular Pharmaceutics School of Pharmaceutical Sciences Peking University Beijing P. R. China; ^3^ Joint Research Institute of Medical and Pharmaceutical Sciences Cheeloo College of Medicine Qilu Hospital of Shandong University Shandong University Jinan P. R. China; ^4^ Peking University‐Yunnan Baiyao International Medical Research Center Beijing P. R. China

**Keywords:** Endosomal escape, Formulation optimization, GLP‐1/FGF21 dual‐agonist mRNA, Insulin sensitization, N‐alkyl phosphoramidate lipids, Obesity, Synergistic therapy, Thermogenesis

## Abstract

Obesity is a complex metabolic disorder associated with an increased risk of type 2 diabetes, cardiovascular diseases, and metabolic dysfunction‐associated steatotic liver disease. While protein‐based therapies like glucagon‐like peptide‐1 (GLP‐1) receptor agonists (e.g., semaglutide) and fibroblast growth factor 21 (FGF21) analogs show promise, their clinical utility is limited by poor tissue targeting and the need for frequent high‐dose injections. To overcome these challenges, we develop an mRNA‐based therapy encoding a long‐acting GLP‐1/FGF21 fusion protein (mGLP‐1/FGF21), engineered with an IgG4 Fc domain to enhance stability. For adipose tissue‐specific delivery, we design a three‐tailed N‐alkyl phosphoramidate lipid (NPL) formulation with enhanced fluidity in lipid‐rich microenvironments, promoting membrane fusion and endosomal escape to significantly improve adipocyte transfection efficiency. Systematic optimization reveals that removing DOPE (while retaining cholesterol) enhanced delivery efficiency 5‐fold compared to conventional four‐component Lipid Nanoparticle (LNP), without inducing metabolic burden or inflammation upon repeated dosing. The optimized platform effectively delivers mGLP‐1/FGF21, demonstrating potent anti‐obesity effects in mice through synergistic GLP‐1 and FGF21 signaling. Treatment induces significant reductions in body weight and fat mass while preserving lean mass and ameliorating hepatic steatosis. By combining adipose‐accumulated mRNA delivery with dual‐hormone therapy, this study presents a novel and localized strategy for obesity treatment.

## Graphical abstract

This study develops an mRNA therapy encoding a long‐acting GLP‐1/FGF21 fusion protein. A three‐tailed ionizable lipid engineered three‐component lipid nanoparticle delivers the mRNA to inguinal adipose tissue. In obese mice, this targeted, dual‐hormone treatment potently reduces body weight, fat mass, presenting a localized strategy for metabolic disease.

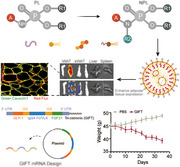

## 1 Introduction

Obesity is a chronic, multifactorial metabolic disorder affecting over 650 million people globally, and is a major driver of type 2 diabetes, cardiovascular disease, and fatty liver disease [1, 2]. While lifestyle interventions and bariatric surgery remain gold‐standard therapies, their limited scalability has spurred the development of pharmacological alternatives. Among these, glucagon‐like peptide‐1 (GLP‐1) receptor agonists [3, 4, 5, 6] and fibroblast growth factor 21 (FGF21) analogs [7, 8] have emerged as leading candidates due to their complementary mechanisms: GLP‐1 suppresses appetite and enhances insulin sensitivity, whereas FGF21 promotes lipid oxidation and thermogenesis. Clinically approved GLP‐1 analogs (e.g., semaglutide, liraglutide) are already widely used for obesity and type 2 diabetes [3, 4, 5, 6], while FGF21‐based therapies (e.g., Pegbelfermin) are under investigation for metabolic disorders, including metabolic dysfunction‐associated steatohepatitis (MASH) [9, 10, 11]. A dual agonist combining these mechanisms could offer superior efficacy by simultaneously targeting energy intake and expenditure.

Despite their promise, the widespread use of GLP‐1 and FGF21 protein drugs remains constrained by several limitations, including poor tissue specificity, proteolytic instability, and the need for frequent high‐dose injections—challenges inherent to peptide/protein delivery. mRNA therapeutics present a compelling alternative by enabling in situ production of therapeutic proteins with tunable, transient, but more durable expression compared to exogenous protein administration [12, 13, 14]. Lipid nanoparticles (LNPs) are the leading platform for mRNA delivery due to their proven clinical efficacy [15, 16, 17]. Nonetheless, current LNP formulations were primarily optimized for hepatic and lymphoid organ delivery, and their performance in non‐hepatic tissues such as adipose depots remains suboptimal [18]. While subcutaneous injection is a practical route to access adipose‐rich areas like inguinal white adipose tissue (iWAT), most commercial LNPs exhibit limited expression in adipocytes due to inadequate cellular uptake. poor adipocyte accumulation, while promoting nonspecific accumulation in lymph nodes or systemic distribution to the liver and spleen [19, 20].

In this study, we addressed the limitations of conventional LNPs by developing a library of N‐alkyl phosphoramidate lipids (NPLs), characterized by a distinct phosphoramidate linker and a three‐tailed structure. The resulting NPL particles exhibited a modulated pKa of 7.5–8.0. This carefully optimized pKa facilitates interaction with extracellular components—specifically glycosaminoglycans overexpressed by adipocytes—while avoiding the significant immunogenicity and restricted distribution of highly cationic LNPs (pKa > 8) or the off‐target drainage to the lymph node, and liver typical of neutral LNPs (pKa 6.3–6.5) [21]. The incorporation of a third hydrophobic tail was designed to augment hydrophobicity and steric bulk, thereby enhancing cellular uptake and membrane fusion. Through systematic optimization, we further streamlined the formulation by identifying and removing redundant helper lipids. We discovered that eliminating DOPE—but not cholesterol—preserved high mRNA delivery efficiency while maintaining metabolic homeostasis in vivo. Furthermore, we engineered an mRNA construct encoding a long‐acting GLP‐1/FGF21 dual agonist. To extend its stability, the construct was fused with either a VLK tag for serum albumin binding or an IgG4 Fc domain for FcRn‐mediated recycling. This dual agonist is designed to synergistically reduce food intake and increase energy expenditure. Collectively, our work presents a tailored mRNA‐LNP platform for adipose‐specific delivery, integrating a rationally designed lipid library with a potent, stabilized dual‐agonist mRNA to achieve enhanced metabolic efficacy and an improved safety profile.

## 2 Results

### 2.1 Hydrophobic‐Branched Chain NPL Improves mRNA Delivery Locally to Fat Tissues Following Inguinal Injection

In our previous study, we identified a cardiolipin‐like di‐phosphoramide lipid that significantly enhanced T cell transfection both in vitro and in vivo without requiring targeting ligands [22]. We also developed an ionizable cationic lipid featuring a piperazine‐centered phosphoramide core (PL), which demonstrated over 60‐fold higher transfection efficiency in the joint cavity upon local administration compared to commercial MC3‐based LNP formulations [23, 24]. While our earlier lipid designs primarily relied on piperazine‐based polyamine structures, this multi‐amine architectures may introduce amine‐related toxicity or metabolic concerns [25, 26], in contrast to the mono‐amine designs commonly used in clinically approved mRNA delivery lipids.

**FIGURE 1:**
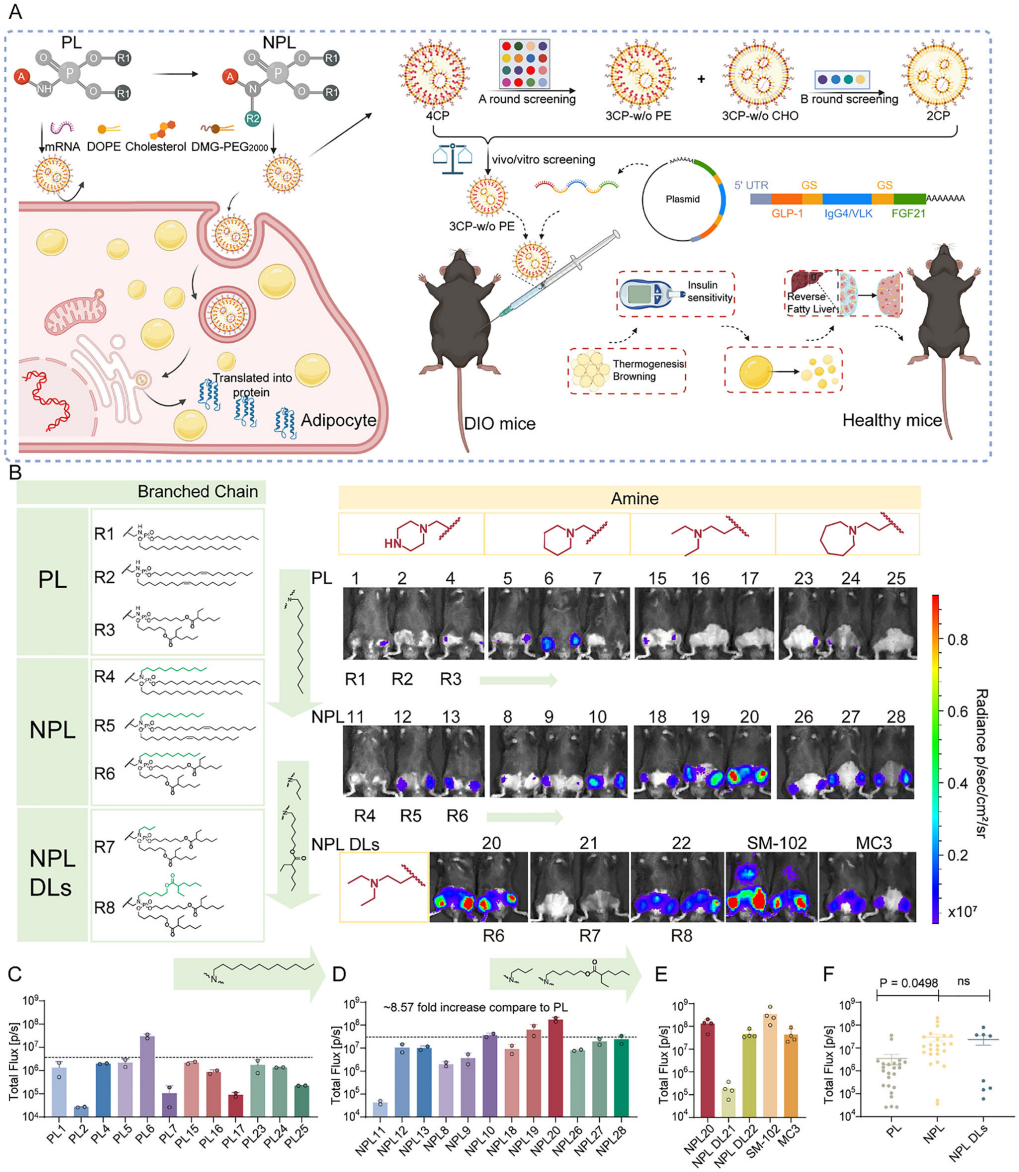
NPL Lipid Structures and the in vivo Transfection in Subcutaneous Adipose Tissue. A) Schematic illustrations of the structures of mono‐amino phosphoramidate lipids (PL) and N‐alkyl phosphoramidate lipids (NPLs) (on the left), and the design of NPL LNPs mediated subcutaneous adipose tissue accumulation (on the right, created with BioRender.com). B) Chemical structures of PL, NPL, and NPL‐DLs lipids and the corresponding mFluc expression mediated by formulated LNPs (0.25 mg/kg) following bilateral subcutaneous administration (*s.c*.) into the inguinal white adipose tissue (iWAT) region of C57BL/6J mice. The mRNA expression levels were recorded and imaged using IVIS at 6 h post‐injection (n = 2–4). C–E) Quantification of the mFluc expression of PL, NPL and NPL‐DLs formulated mFluc LNPs. F) Quantitative comparisons of mFluc mRNA expression delivered by PL, NPL, and NPL‐DLs. Data are represented as mean ± SD. For F, Statistical significance is calculated via a two‐way ANOVA with a Tukey's multiple comparisons test.

Motivated by this distinction, we designed a new class of mono‐amine‐based phosphoramide lipids. Using a combinatorial approach, we integrated three types of hydrophobic tails—saturated alkyl chains, unsaturated alkenyl chains, and branched ester chains—with different amino head structures (including their N‐alkyl derivatives), resulting in a library of 24 distinct lipids. The synthesis of these mono‐amino phosphoramide lipids proceeded via a two‐step nucleophilic substitution of phosphorus oxychloride (POCl_3_). In the first step, two hydrophobic tails (R1) replaced two chlorine atoms in POCl_3_ to form an O‐alkyl intermediate. The second step involved reaction with either primary amines or functionalized secondary amines, yielding phosphoramidate lipids (PL) or N‐alkylphosphoramidate lipids (NPL) with tertiary amines, respectively (Figure 1A; Scheme S1). The structures of all synthesized lipids were confirmed by NMR spectroscopy. For the lead candidates PL16, PL17, NPL19, and NPL20, the corresponding ^1^H NMR, ^13^C NMR, and mass spectra are provided in Figures S1–S12. LC–MS analyses confirmed purities of >90% for these compounds (Figure S13). Furthermore, the successful formation of the N‐alkyl phosphoramidate moiety was confirmed by HMBC NMR spectroscopy (Figure S14).

To evaluate the effect of N‐alkyl modifications on mRNA delivery efficiency and identify lead ionizable lipid candidates, we prepared LNP formulations containing ionizable NPL lipids combined with helper lipids (DOPE, cholesterol, and DMG‐PEG2000) at a standard molar ratio of 35:16:46.5:2.5 (Figure 1A). These LNPs, encapsulating firefly luciferase mRNA (mFluc), demonstrated 50–80% encapsulation efficiency (except PL1, NPL11, NPL18) and mostly maintained particle sizes of 80–200 nm (Table S1).

For in vivo transfection assessment, LNPs were administered subcutaneously near iWAT, with luminescence signals quantified 6 h post‐injection via IVIS imaging (Figure 1B). Structural analysis revealed that N‐alkyl modifications—where an additional hydrophobic tail (R2) was conjugated to the phosphoramide secondary amine—consistently enhanced local protein expression compared to two‐tailed PL analogs, independent of amino head group structure (Figure 1B–D). Among all N‐alkyl derivatives (NPL and NPL‐DLs), lipids with linear amino head groups (NPL 18–20) generated the strongest luminescence signals (Figure 1E,F).

We further investigated the structural determinants of transfection efficiency by systematically varying the N‐alkyl chain length and biodegradability. Key findings demonstrated that replacement of the optimal 12‐carbon (12C) N‐alkyl chain with either shorter 4‐carbon (4C) chains or branched ester linkages markedly diminished protein expression, with the 4C variant exhibiting the most significant reduction (Figure 1B,E). These data establish two critical requirements for optimal performance: i) N‐alkyl chains ≥12 carbons in length, and ii) O‐alkyl‐branched hydrophobic tails. Notably, lead candidate NPL20 mediated fivefold higher expression than MC3 while matching SM‐102's efficacy, while showing preferential adipose tissue accumulation rather than the hepatic distribution characteristic of SM‐102 (Figure 1E).

### 2.2 pKa and Membrane Fluidity Impact the mRNA Delivery Efficiency of NPL20 to Adipose Tissue

**FIGURE 2:**
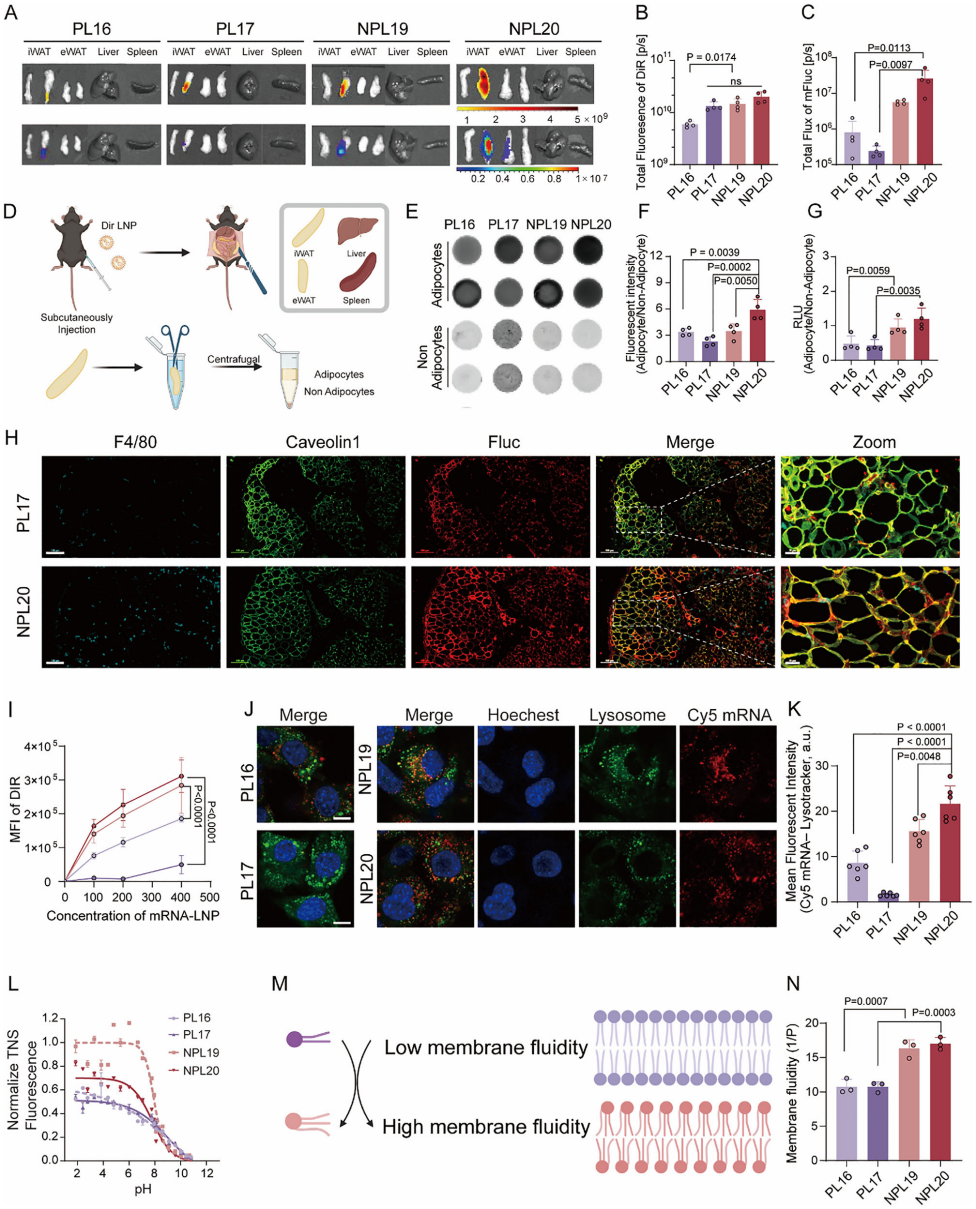
Expression and Distribution of NPL20 LNPs (mFLuc) in C57BL/6J Mice. A) 1% DiR‐labelled PL16, PL17, NPL19 and NPL20 mFluc LNPs (0.15 mg/kg) were subcutaneously injected (*s.c*.) in the inguinal white adipose tissue (iWAT) region of C57BL/6J mice and IVIS determination of tissue expression (mFluc RNA) and distribution (DiR) at 6 h post‐injection (group n = 4, only 1 representative was presented). The unit of fluorescent scale bar is 6 × 10^8^–5 × 10^9^ photons/sec/cm^2^/sr. The radiance scale of 1 × 10^5^–1 × 10^7^ photons/sec/cm^2^/sr. B,C) Quantification fluorescent signal of DiR distribution (B) and Fluc expression (C) delivered by PL16, PL17, NPL19 and NPL20 LNPs. D) Schematic of the separation of adipocytes and non‐adipocytes from white adipose tissue (created with BioRender.com). E) The distribution of PL16, PL17, NPL19, and NPL20 LNPs in adipocytes and non‐adipocytes of iWAT in per cell count. The fluorescence image of DiR visualized by Typhoon, n = 4, only 2 representative was presented. F) Quantitative comparison of fluorescent signals between adipose and non‐adipose tissue samples in E. G) Quantitative comparison of Fluc expression in iWAT in per cell count, n = 4. H) Colocalization of luciferase protein（red colour） with F4/80 (staining for macrophage, cyan color), caveolin‐1 (staining for adipocyte cell membrane, green colour) in the iWAT. Representative data in C were independently repeated three times with similar results. Scale bar = 100 µm, zoom scale bar = 20 µm I) Uptake of PL16, PL17, NPL19 and NPL20 LNPs containing 1% DiR with different concentrations in 3T3‐L1 cells, n = 4, analyzed by flow cytometry. J,K) Confocal imaging of the intracellular distribution of PL16, PL17, NPL19, and NPL20 encapsulating Cy5 labelled RNA LNPs in 3T3‐L1 cells, n = 6. Scale bar = 8 µm. Data are represented as mean ± SD. L) Titration curves of pKa for PL/NPL16‐20 (n = 2). M,N) Membrane fluidity of PL/NPL LNPs, n = 3. For B, C, F, G and K, statistical significance was calculated via ordinary one‐way ANOVA, followed by Tukey's multiple comparisons test. For I and N, statistical significance was calculated via two‐way ANOVA, followed by Tukey's multiple comparisons test (I) and Šídák's multiple comparisons test (N) calculated via Mann‐Whitney *U* test.

NPL20 was selected as the lead ionizable lipid due to its consistently high in vivo mRNA expression. To assess its biodistribution and transfection efficiency in adipose tissue, we administered DiR‐labeled LNPs encapsulating mFluc RNA to C57BL/6J mice. As controls, we included the non‐N‐alkyl PL lipids PL16 (oleyl O‐alkyl tails) and PL17 (degradable branched O‐alkyl tails), as well as the N‐alkyl lipid NPL19 (oleyl O‐alkyl tails). NPL20 LNPs demonstrated preferential distribution and enhanced mRNA expression in adipose tissues (Figure 2A). While LNP accumulation in adipose tissue was∼1.4‐fold higher than that of PL17 and NPL19 LNPs and ∼3.4‐fold higher than PL16, the mRNA expression levels were nearly 12‐fold greater compare to average of PL/NPL16‐19, suggesting that both improved cellular uptake and enhanced endosomal escape contribute to NPL20's superior performance (Figure 2A–C).

To further determine the cellular targets of mRNA expression within adipose tissue for different LNPs, we enzymatically dissociated iWAT using collagenase to separate adipocytes from non‐adipocytes (Figure 2D). Quantitative fluorescence imaging using the Typhoon system demonstrated broad lipid distribution across both cell types, while adipocytes exhibiting 2‐ to 6‐ fold higher signal intensity than non‐adipocytes across all different LNP groups (Figure 2E,F). Notably, NPL20 showing ∼2 fold greater accumulation in adipocytes compared to other LNPs, confirming preferential lipid uptake in adipocytes (Figure 2E,F). Bioluminescence analysis further revealed that both adipocytes and non‐adipocytes expressed the luciferase reporter. However, N‐alkyl‐modified lipids (NPL19 and NPL20) significantly increased the adipocytes/non‐adipocytes expression ratio (∼2.4 fold greater) compared to unmodified lipids (PL16 and PL17) (Figure 2G). These results suggest that N‐alkylation not only improves overall expression but also selectively enhances delivery to adipocytes (Figure 2F,G). Further validation by fluorescent microscopy showed that luciferase expression of NPL20 LNPs was distributed throughout the iWAT tissue, unlike PL17, which was low and restricted to surface regions. Co‐localization analysis with the adipocyte marker caveolin‐1 confirmed the intracellular luciferase expression in mature adipocytes, as evidenced by green‐to‐yellow merged signals (Figure 2H).

To explore the rationale causing improved adipocyte accumulation and expression for NPL20, we evaluated the cellular uptake and endosomal escape efficiency of LNPs in matured adipocytes in vitro. Specifically, DiR‐labeled LNPs were used to quantify cellular uptake, while Cy5‐labeled mRNA was employed to assess endosomal escape capability. The results indicated that N‐alkyl‐modified lipids (NPL19 and NPL20) significantly enhanced cellular lipid uptake in a dose‐dependent manner compared to their unmodified counterparts (PL16 and PL17) (Figure 2I). Similarly, confocal microscopy revealed that NPL19 and NPL20 exhibited approximately 4‐fold higher intracellular fluorescence intensity compared to PL16 and PL17, highlighting the role of N‐alkyl‐modified lipids (NPL19 and NPL20) in facilitating cellular delivery (Figure 2J,K). This improvement may be attributed to the reduction in apparent pKa from 9.42/9.05 (PL16/PL17) to 7.92/7.78 (NPL19/NPL20) (Figure 2L; Table S2). The pKa value is critical for controlling adipocyte accumulation. Mono‐amino‐PL lipids (PL16/17), with their higher pKa, retain a positive charge under physiological conditions. This cationic charge likely promotes binding to local collagen following injection, which limits deeper penetration and distribution within the adipose tissue (Figure 2A,H). Conversely, lipids with a lower pKa (e.g., SM‐102, pKa 6.3–6.5) are neutrally charged and prone to systemic drainage, leading to accumulation in the liver and other organs (Figure 1B). The optimized pKa range of 7.5–8.0 was therefore found to maintain a balance—facilitating adipocyte accumulation through interaction with surface glycosaminoglycans [21], while still allowing for broad adipose tissue distribution and limited systemic exposure.

Since membrane fusion is critical for endosomal escape, we further measured fusion capacity using fluorescence polarization assays. The N‐alkyl‐modified lipids (NPL19 and NPL20) significantly enhanced membrane fluidity (Figure 2M,N), correlating with their improved escape performance. We further investigated the reason for causing improved membrane fusion using molecular modeling method. We find structural N‐alkyl modification resulted in wider bond angles between P─N bond and N‐branched chain bond while elongated their bond lengths, potentially contributing to the improved lipid packing flexibility (Figure S15). Together, these findings suggest that N‐alkyl modification lowers the pKa and increases bond angle and lengths to improve both adipocyte accumulation and endosomal escape. Additionally, confocal microscopy revealed that NPL20 (N‐alkyl modification with degradable branched O‐alkyl) exhibited more rapid release of mRNA from endosomes than NPL19 (Figure 2J,K). These findings are consistent with the superior expression performance exhibited by NPL20.

### 2.3 Optimization of LNP Composition for Metabolic Applications

**FIGURE 3:**
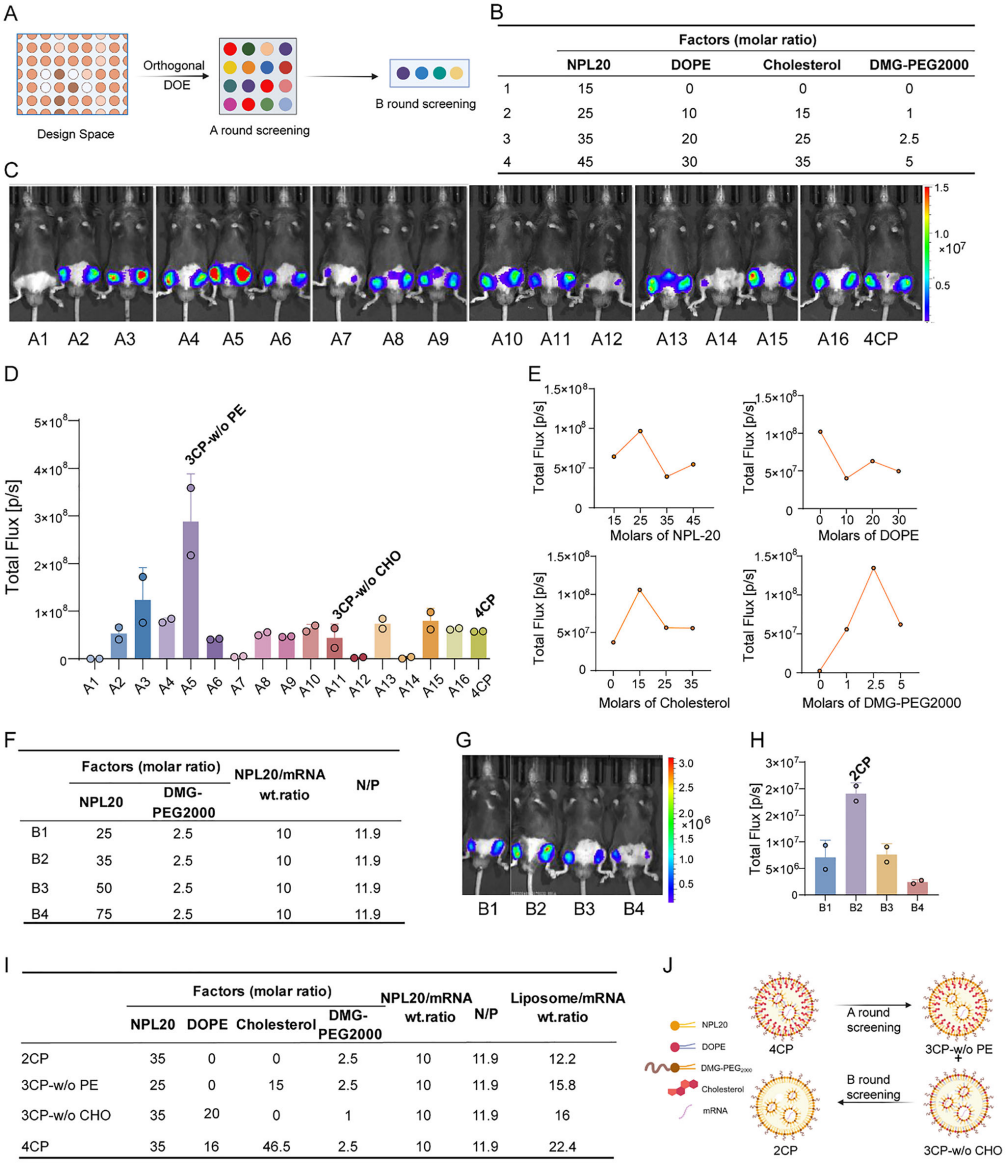
Formulation Screening for Simplified LNP Design. A) Schematic illustration of two round screenings (created with BioRender.com). B) Orthogonal screening of LNP formulations with four levels. C,D) Expression of mFluc and quantification at 6 h after subcutaneous injection (*s.c*.) in the inguinal white adipose tissue (iWAT) region with orthogonal screened LNPs, the original 4CP (0.15 mg/kg, n = 2, 1 mouse). E) The impacts of four individual components on the luminescence intensity, each data point represents the average relative luminescence of the four LNP formulations with the given excipient molar ratio. F) B round screening of LNP formulations with two components. G,H) Expression of mFluc and quantification at 6 h after *s.c*. in the inguinal white adipose tissue (iWAT) region with B round screening LNPs (0.15 mg/kg, n = 2, 1 mouse). I) Formulation details of the reformulated LNPs and the original 4CP. J) Schematic illustration of two round screening's results (created with BioRender.com). Data in (D) and (H) presented as mean ± SD.

Given the potential impact of exogenous lipids on metabolic homeostasis in obesity, we sought to develop simplified LNP formulations with reduced lipid complexity while maintaining delivery efficiency. Through systematic screening, we progressively evaluated the simplified compositions (Figure 3A). An orthogonal assay was conducted in the first round (round A), where we maintained a constant N/P ratio while systematically varying the molar ratios of the four components (Figure 3B; Table S3). We observed that PEG‐free formulations (A1/A7/A12/A14) yielded unstable LNP with diameters exceeding 1000 nm and negligible expression (Figure 3C; Table S4). Most of other formulations exhibited a similar particle size between 100–200 nm with PDIs below 0.3 and encapsulation efficiency (EE) between 50–80%, confirming homogeneous LNP formation (Table S4). Compared to conventional four‐component LNP (4CP), we identified A5 as the optimal DOPE‐free three‐component LNP (3CP‐w/o PE), demonstrating approximately 5‐fold higher expression (Figure 3C,D). Meanwhile, A11 emerged as the optimal cholesterol‐free three‐component LNP (3CP‐w/o CHO), exhibiting comparable expression to 4CP while achieving a lower liposome/mRNA ratio (Table S3). Further, by analyzing luminescence in relation to the molar ratios of individual excipients, we identified lower DOPE ratios while moderate ratios of NPL20, cholesterol and PEG exhibited higher expression (Figure 3E).

The formulations were further optimized in the second screening round (round B) to investigate two‐component LNP composed solely of NPL20 and PEG. We maintained a constant N/P ratio and fixed the PEG molar ratio at 2.5% while varying the molar ratios of NPL20 from 25% to 75% (Figure 3F; Table S5). The B‐round formulations exhibited a particle size varying from 103.7 to 206.2 nm with PDIs below 0.3 and EE between 70–80% (Table S6). The NPL20/PEG (35%/2.5% molar ratio) formulation exhibited optimal expression and was identified as the lead two‐component LNP (2CP) (Figure 3G,H). Through two rounds of screening, we identified 3CP‐w/o PE, 3CP‐w/o CHO, and 2CP as the reformulated LNP which enabled a lower liposome/mRNA ratio with simplified lipid diversity (Figure 3I,J). Furthermore, we assessed the stability of 2CP, 3CP‐w/o PE, and 4CP systems over 28 days at both −80°C and 4°C. All formulations maintained stable physicochemical properties and protein expression levels at 4°C, comparable to commercial SM‐102. Notably, 3CP‐w/o PE also exhibited excellent stability at −80°C, while 3CP‐w/o CHO and 2CP showed increased particle size and reduced expression under the same condition. These results confirm that 3CP‐w/o PE combines simplified composition with promising delivery efficiency and improved stability (Figure S17). We next evaluated whether the 3CP‐w/o PE formulation could be extended to other PL and NPL lipids. The resulting LNPs exhibited transfection efficiency that was higher than or comparable to traditional 4CP systems, establishing 3CP‐w/o PE as a versatile, simplified alternative to conventional formulations (Figure S18).

The pKa of the simplified LNP formulations was then determined using both TNS binding assays and electrophoretic mobility measurements (zeta potential, ZP) (Figure S19). The ZP‐titration curve occurred over a broader pH range than the TNS‐based curve, and the calculated pKa from ZP was slightly lower (by ∼0.5 units). This discrepancy is consistent with previous findings [27], as ZP measures the net charge of the entire particle, while TNS primarily detects the charge in the lipid membrane surface. Despite the difference in absolute values, the trends from both measurements were similar. Formulation 2CP showed the lowest pKa, whereas 3CP‐w/o CHO showed the highest. This suggests that 2CP may have more negatively charged mRNA packed on its surface, while 3CP‐w/o CHO likely presents more ionizable lipids. Furthermore, the broader pH transition of 2CP suggests uneven charge distribution across the LNP population, which could explain its decreased stability and lowest expression level. In contrast, both 3CP‐w/o PE and 4CP exhibited comparable pKa values. A pKa of ∼7.5 confers a slight positive charge, which may promote adipocyte accumulation while still permissive for adipose tissue diffusion.

**FIGURE 4:**
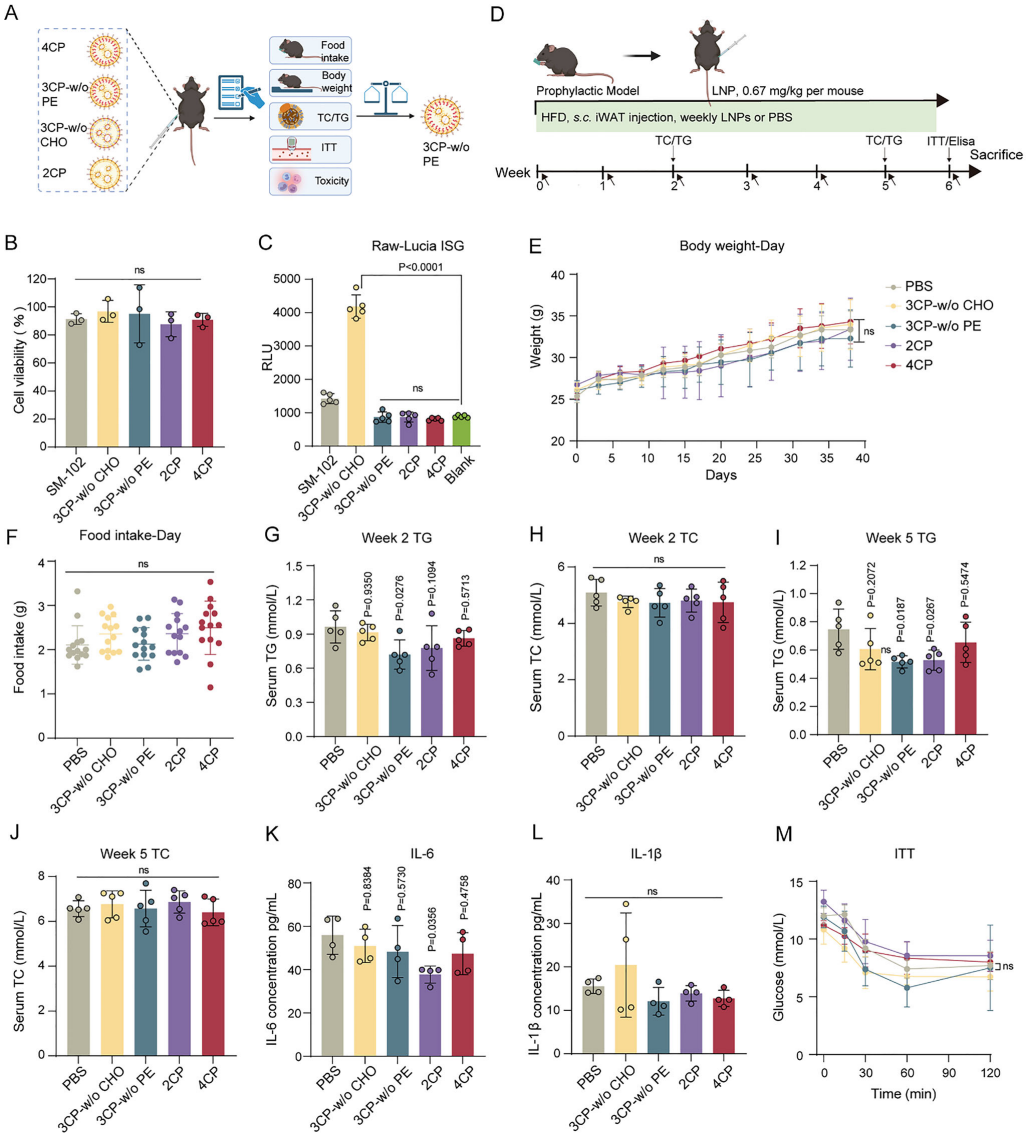
Comparisons of the Metabolic Impacts of the Simplified LNPs after Multiple Injections in Preventive DIO Mice. A) Schematic of systematic evaluation of safety, and metabolic effects of simplified LNP formulations (4CP, 3CP‐w/o PE, 3CP‐w/o CHO, 2CP, created with BioRender.com). B) Viability of 3T3‐L1 cells treated with simplified LNP and 4CP for 48 h (n = 3 biologically independent wells). C) IFN‐I induction of simplified LNPs, 4CP (0.1 µg mRNA/well) in RAW‐Lucia ISG cells after 24 h incubation. SM‐102 LNPs were set as a positive control (n = 5 biologically independent wells). D) Schematic of the experimental design (created with BioRender.com). Male mice were subcutaneously injected (*s.c*.) in the inguinal white adipose tissue (iWAT) region with different formulations of NPL20 LNPs without mRNA (equivalent to 0.67 mg/kg NPL20 mRNA LNPs) or PBS weekly since the beginning of HFD feeding. E) Body weight curve during six‐week treatment (n = 5 per group). F) Average food intake per mouse per day during continuous HFD feeding periods. G,H) Serum TG and TC levels in HFD mice at week 2 (n = 5 per group). I,J) Serum TG and TC levels in HFD mice at week 5 (n = 5 per group). K,L) Serum IL‐6 and IL‐1β levels in HFD mice at week 6 (n = 4 per group). M) Insulin tolerance test (ITT) at 6 weeks of treatment (n = 4 per group). Data represented as mean ± SD. For B, C and G–L, statistical significance is calculated via an ordinary one‐way ANOVA with a Dunnett's multiple comparisons test. For E, F and M, statistical significance is calculated via a two‐way ANOVA with a Tukey's multiple comparisons test.

We further conducted a systematic comparative evaluation of the delivery efficiency, safety profile, and metabolic impacts of the simplified LNP formulations (w/o RNA) (Figure 4A). Cytotoxicity evaluation in 3T3‐L1 cells by MTT and LDH assays demonstrated comparable cell viability among simplified LNPs (2CP, 3CP), conventional 4CP and commercial SM‐102 formulations, confirming their biocompatibility (Figure 4B; Figure S20). Immunogenicity testing in RAW‐Lucia ISG cells showed that, except for 3CP‐w/o CHO—potentially due to its higher zeta potential (10.13 mV)—all reformulated LNPs exhibited lower immunogenicity than SM‐102 LNPs (Figure 4C).

To investigate the effects of various simplified LNPs on metabolic programming, we established a prophylactic model in which mice on a 60% high‐fat diet (HFD) received weekly subcutaneous inguinal injections of blank LNPs (Figure 4D). It is noteworthy that during the 6‐week treatment period, none of the LNP formulations demonstrated significant body weight changes compared to the PBS control group (Figure 4E). Food intake remained stable across all groups (Figure 4F), indicating that the tested formulations did not exhibit apparent lipotoxicity. Early intervention (Week 2) revealed that all LNP‐treated groups had reduced serum triglyceride (TG) levels compared to PBS, though total cholesterol (TC) remained unchanged (Figure 4G,H). The 3CP‐w/o PE group showed the most pronounced effect, achieving ∼25% greater TG reduction. By mid‐term (Week 5), TG‐lowering efficacy further improved, with 3CP‐w/o PE exhibiting a ∼30% greater reduction than PBS (Figure 4I), while TC levels remained unchanged (Figure 4J), This progressive TG reduction correlated with improved adipocyte function, suggesting that PE lipids may facilitate TG synthesis, whereas PE‐depleted LNPs attenuate it—consistent with prior reports implicating phosphocholine and phosphoethanolamine metabolism regulates thermogenesis [28, 29, 30, 31].

Further, systemic inflammation was evaluated and the LNP treatment groups demonstrated lower serum IL‐6 and IL‐1β levels compared to PBS group (Figure 4K,L), which is induced by obesity. However, we noticed that 2 out of 3CP‐w/o CHO group showed elevated IL‐1β secretion, consistent with in vitro evaluation (Figure 4C) [32]. Metabolic safety was evaluated at study endpoint through insulin tolerance test (ITT). Results showed that the 3CP‐w/o PE group maintained normal insulin and glucose tolerance without adverse effects relative to other treatment groups, confirming the metabolic safety of the 3CP‐w/o PE formulation (Figure 4M).

Our comparative data demonstrate that the simplified 3CP‐w/o PE LNP formulation achieves potent mRNA delivery while exhibiting an optimal safety profile for chronic obesity intervention. Notably, it showed no inflammatory activation, HFD‐associated weight gain, dysregulated serum TG, or other metabolic perturbations. Based on its combined mRNA delivery efficiency and metabolic neutrality, we selected 3CP‐w/o PE for further development.

### 2.4 GLP‐1/FGF21 mRNA in NPL20 LNPs Prevents Fat Accumulation in DIO Mice

Glucagon‐like peptide‐1 (GLP‐1) and fibroblast growth factor 21 (FGF21) serve as critical metabolic regulators that act through a multi‐layered synergistic network in adipose tissue to optimize metabolic homeostasis [33, 34]. GLP‐1 enhances insulin sensitivity while simultaneously stimulating preadipocyte differentiation [35, 36, 37, 38]. Complementing these actions, FGF21 drives mitochondrial oxidative function through the AMPK–SIRT1–PGC1α pathway and activates the PGC1α‐UCP1 thermogenic cascade [39, 40, 41]. Importantly, FGF21 potentiates insulin signaling [42], creating a robust synergistic effect with GLP‐1. Furthermore, GLP‐1 enhances adipose tissue responsiveness to FGF21 by upregulating FGFR1 expression [43]. Together, this multi‐targeted regulatory system not only suppresses pathological lipolysis but also enhances energy expenditure.

**FIGURE 5:**
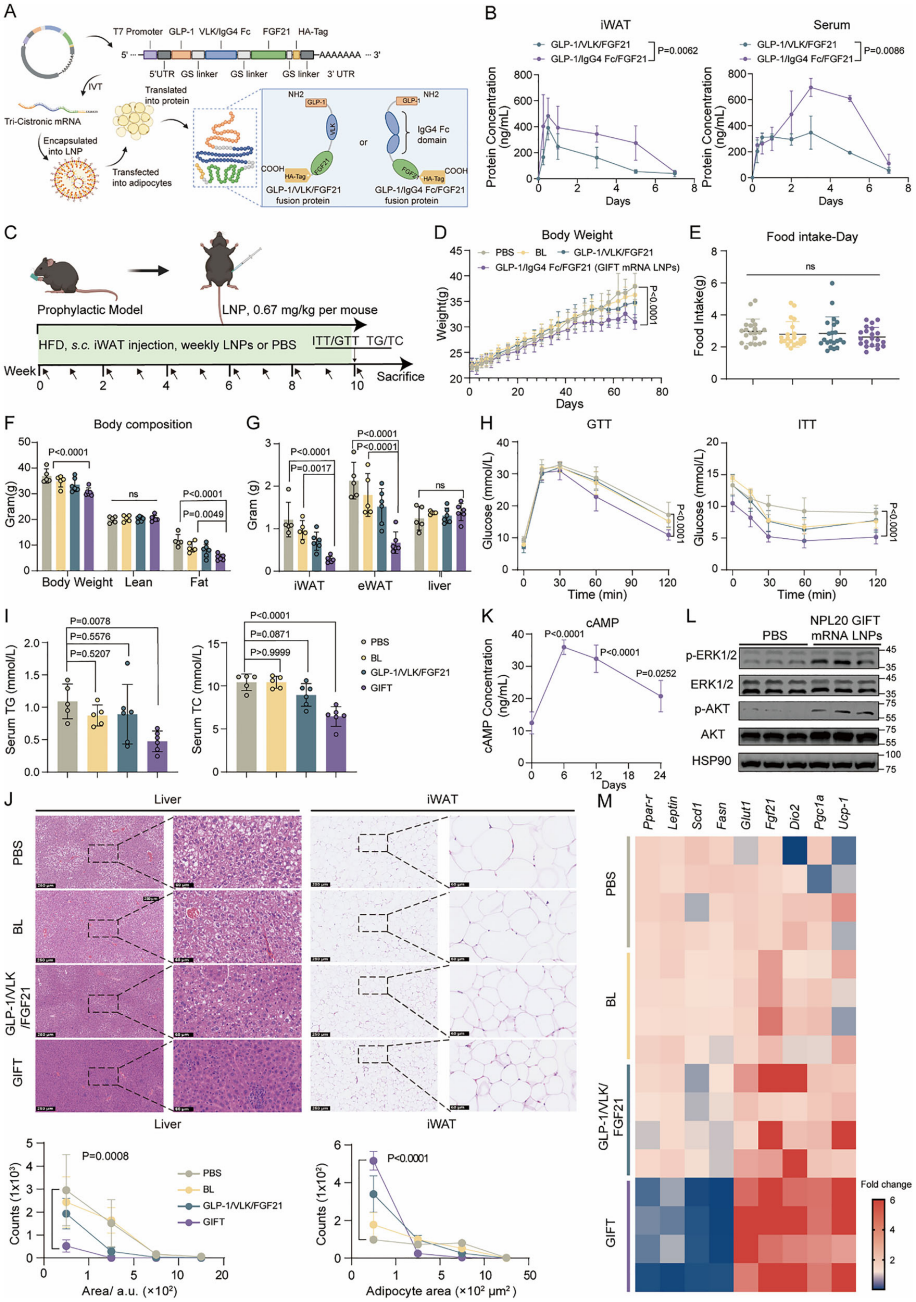
GLP‐1/IgG4 Fc/FGF21 Tri‐cistronic (GIFT) mRNA in NPL20 LNPs Prevents Fat Accumulation in DIO Mice. A) Schematic of the mRNA sequence design and therapeutic delivery strategy (created with BioRender.com). B) Pharmacokinetic profile of GLP‐1/VLK/FGF21 and GIFT mRNA expression in iWAT and serum following a single dose (0.67 mg/kg mRNA, n = 3). C) Schematic of the experimental design for prophylactic model (created with BioRender.com). Male mice were subcutaneously injected (*s.c*.) in the inguinal white adipose tissue (iWAT) region with NPL20 LNPs without/with mRNA (6.7 mg/kg NPL20 BL LNPs, equivalent to 0.67 mg/kg NPL20 mRNA LNPs) or PBS weekly since the beginning of HFD feeding. D) Body weight curve during ten‐week treatment (n = 5–6 per group). E), Average food intake during continuous HFD feeding periods. F) Body composition (n = 5–6 per group) at 10 weeks of treatment. G) Tissues weight (n = 5–6 per group) at sacrifice. (H) Glucose tolerance test (GTT) and insulin tolerance test (ITT) at 10.5 and 11.5 weeks of treatment, respectively (n = 5–6 per group). I) Serum TG and TC levels in DIO mice at sacrifice (n = 5–6 per group). J) H&E staining of iWAT and liver, and the size distribution of adipocytes in iWAT and lipid droplets in liver. K) cAMP levels in iWAT at indicated times post LNP injection (0.67 mg/kg, n = 4). cAMP is a key downstream signaling molecule of the activated GLP‐1/GLP‐1R pathway. L) Western blot analysis of ERK and AKT phosphorylation. Phosphorylation of these kinases indicates activation of the downstream FGF21 signaling pathway. M) Analysis of mRNA levels for lipogenic, pan‐adipocyte, and browning relative genes in iWAT from DIO mice after ten‐week NPL20 LNPs treatment. Data are represented as mean ± SD. For B statistical significance is calculated via a Mixed Model with a Sidak's multiple comparisons test. For D–H, J, statistical significance is calculated via a two‐way ANOVA with a Tukey's multiple comparisons test. For I and K statistical significance is calculated via a one‐way ANOVA with a Dunnett's multiple comparisons test.

To exploit this synergistic relationship, we designed an mRNA sequence encoding a fusion protein incorporating both GLP‐1 and FGF21, along with either a VLK or IgG4 Fc domain for pharmacokinetic (PK) and stability optimization. The VLK domain improves stability through serum albumin binding, while the IgG4 Fc domain mediates FcRn receptor engagement to reduce proteolytic degradation. In our constructs, an FGF21 variant (L98R, P171A, S167H, R175L) with enhanced β‐klotho binding affinity was fused to either VLK or IgG4 Fc at its N‐terminus, while a GLP‐1 analog was connected via GS linkers to the N‐terminus of the IgG4 Fc/VLK‐FGF21 components, creating dual‐target fusion proteins (Figure 5A) [34]. Using NPL20 LNPs, we delivered these two GLP‐1/FGF21 tri‐cistronic mRNA constructs, differentiated by either a VLK or an IgG4 Fc linker, first to 3T3‐L1 adipocytes in vitro, where Western blot confirmed robust fusion protein expression (Figure S21). We then administered a single subcutaneous injection of each mRNA construct (0.67 mg/kg of mRNA) into the inguinal region of HFD mice. Analysis of protein levels revealed that the IgG4 Fc‐linked construct produced superior results in vivo (Figure 5B). It demonstrated robust expression and accumulation within iWAT, with an area under the curve (AUC_0‐t_) ∼2 fold‐greater and a higher Cmax than the VLK‐linked construct (Figure 5B; Table S7). Furthermore, the IgG4 Fc fusion protein exhibited an enhanced systemic exposure, yielding a ∼2‐fold greater systemic AUC_0‐t_ than the VLK group. Overall, the IgG4 Fc linker enables efficient and sustained expression.

We next evaluated the prophylactic potential of mRNA encoding the two fusion proteins in a HFD induced obesity model through a ten‐week prevention regimen (Figure 5C). One week dosing interval was selected based on the PK profile of the expressed protein. Mice treated with NPL20 LNPs containing GLP‐1/FGF21 mRNA demonstrated significantly enhanced anti‐obesity effects compared to empty LNP controls. The treatment group exhibited reduced food intake, slower body weight gain, and significant decreases in adipose tissue mass. Consistent with the PK profiles of the two types of mRNA, the GLP‐1/IgG4 Fc/FGF21 mRNA‐treated group showed approximately 78% lower iWAT weight and over 65% lower in epididymal white adipose tissue (eWAT) mass compared to the PBS‐treated group (Figure 5D–G), more prominent than the VLK group. We therefore, designated the optimized mRNA construct GLP‐1/IgG4 Fc/FGF21 Tri‐cistronic mRNA as GIFT mRNA. Furthermore, GIFT mRNA LNP treated mice exhibited improved insulin sensitivity (Figure 5H) and marked reductions in serum total TC and TG, indicating amelioration of dyslipidemia (Figure 5I).

Histological examination revealed significant tissue‐level improvements. iWAT showed attenuated adipocyte hypertrophy, demonstrating enhanced adipose tissue remodeling (Figure 5J). Concurrently, liver sections from treated mice exhibited substantially reduced lipid droplet accumulation, indicating reversal of hepatic steatosis (Figure 5J). These findings suggest that GIFT mRNA therapy induces systemic metabolic reprogramming capable of counteracting obesity‐related pathophysiology. Repeated administration of GIFT mRNA LNP was well‐tolerated with no adverse effects observed in major organs. This was supported by liver and kidney function parameters, which showed no significant differences from the PBS control group. Notably, serum creatinine levels were significantly lower in the LNP group, suggesting a potential improvement in renal function (Figure S22).

To elucidate the molecular mechanism by which GIFT mRNA reprograms adipose tissue, we assessed the activation of its target pathways. GLP‐1 receptor engagement was confirmed by a robust, time‐dependent increase in cAMP levels, indicating potent agonist activity (Figure 5K). Similarly, FGF21 pathway activation was demonstrated by enhanced phosphorylation of its key downstream effectors, ERK and AKT, which are known to be triggered by the FGFR1c/β‐Klotho complex (Figure 5L). At the transcriptional level, quantitative PCR demonstrated significant upregulation of thermogenic genes, including *Ucp1* (11.8‐fold), *Pgc1α* (3.9‐fold), and *Dio2* (12.3‐fold), consistent with FGF21's known activation of the PGC1α‐UCP1 pathway. Concurrently, we observed an 90–95% reduction in mRNA levels of lipogenic enzymes *Fasn* and *Scd1*, aligning with the synergistic activation of AMPK pathways by GLP‐1 and FGF21 (Figure 5M) [39, 44, 45]. These coordinated changes in gene expression demonstrate the tri‐fusion protein's ability to comprehensively modulate metabolism by simultaneously enhancing energy expenditure and suppressing lipid accumulation.

### 2.5 GIFT mRNA LNPs Exhibits Superior Anti‐Obesity Effects

**FIGURE 6:**
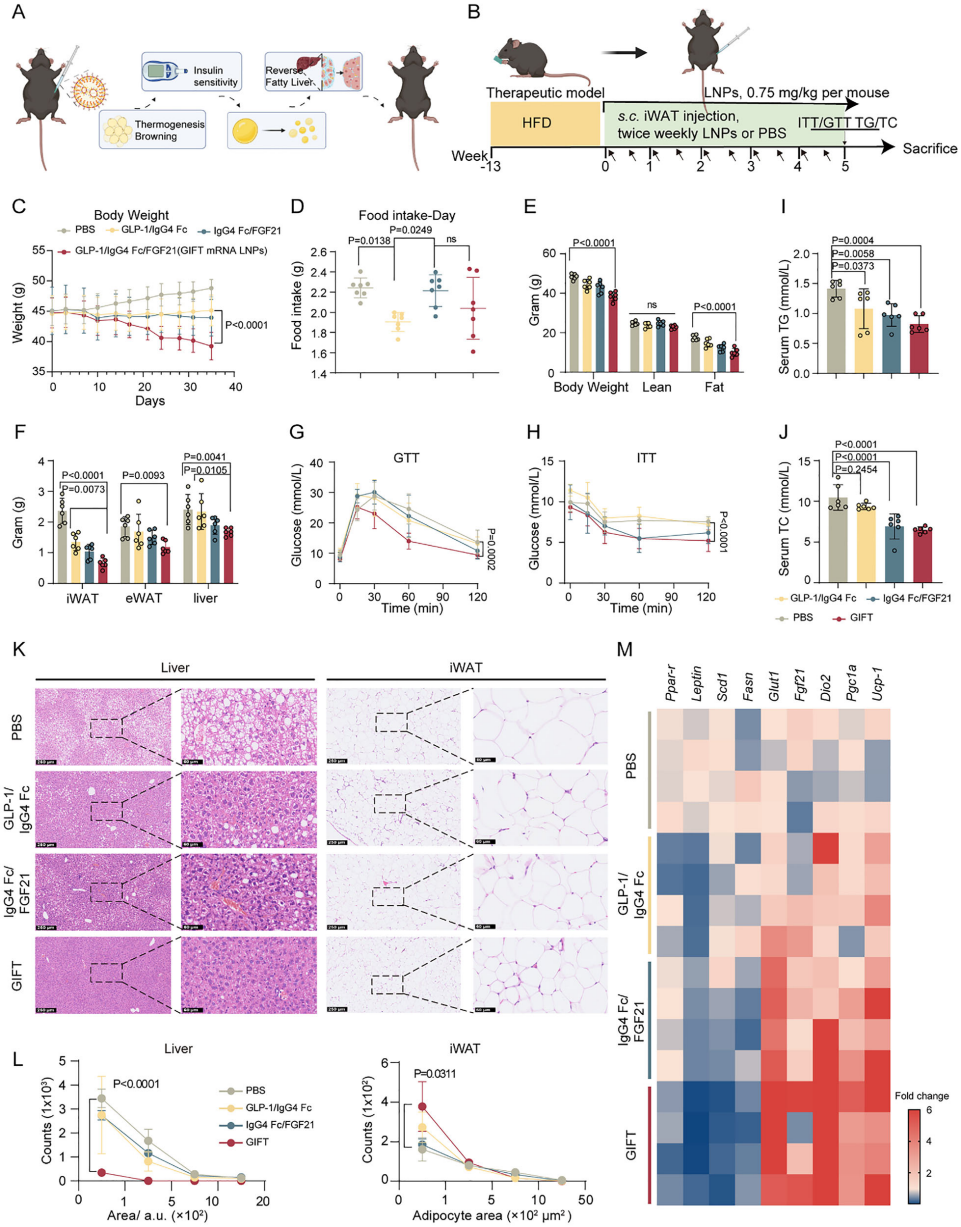
GLP‐1/IgG4 Fc/FGF21 Tri‐cistronic (GIFT) mRNA Exhibits Superior Anti‐obesity Effects. A) Schematic illustration of the therapeutic evaluation of GIFT mRNA in diet‐induced obese (DIO) mice (created with BioRender.com). B) Schematic of the therapeutic model experimental design (created with BioRender.com). Male mice were subcutaneously injected (*s.c*.) in the inguinal white adipose tissue (iWAT) region with NPL20 LNPs encapsulating mRNA (0.75 mg/kg NPL20 mRNA LNPs) or PBS (control) twice weekly, starting after 13 weeks of high‐fat diet (HFD) feeding. C) Body weight of DIO mice post administration of NPL20 LNPs with three mRNAs before disturbance by metabolic measurements. D) Average daily food intake per mouse within one week after the first administration. E) Body composition measured by EchoMRI. F) Tissues weight at sacrifice. G,H) Glucose tolerance test (GTT) and insulin tolerance test (ITT). I,J) Serum triglyceride (TG) and total cholesterol (TC) level in DIO mice at sacrifice. K,L) H&E staining of iWAT and liver (K), and corresponding size distribution of adipocytes in iWAT and the lipid droplets in liver (L), n = 3 per group, Scale bar represents 250 µm. M) Analysis of mRNA levels for lipogenic, pan‐adipocyte, and browning relative genes in iWAT from DIO mice after ten‐week NPL20 LNPs treatment (n = 4 per group). Data are represented as mean ± SD. For C–H and L, statistical significance is calculated via a two‐way ANOVA with a Tukey's multiple comparisons test. For I and J, statistical significance is calculated via a one‐way ANOVA with a Dunnett's multiple comparisons test.

Building on the demonstrated advantages of IgG4 Fc in our prophylactic model, we next investigated the therapeutic potential of GIFT mRNA in a diet‐induced obesity (DIO) mouse model. To rigorously assess the synergistic anti‐obesity effects, we designed two single‐target control mRNAs: GLP‐1/IgG4 Fc and IgG4 Fc/FGF21, both utilizing IgG4 Fc to improve stability (Figure 6A). This comparative approach allowed us to determine whether the tri‐cistronic construct offered superior efficacy to either monotherapy alone.

DIO mice received localized injections of NPL20 LNPs encapsulating the respective mRNAs into iWAT twice weekly for five weeks (Figure 6B). The tri‐cistronic mRNA demonstrated significant therapeutic advantages, inducing a 20% reduction in body weight, whereas both single‐target mRNAs merely prevented further weight gain (Figure 6C). While GLP‐1/IgG4 Fc mRNA showed the most pronounced suppression of food intake—consistent with GLP‐1's established anorexigenic effects (Figure 6D)—the tri‐cistronic construct produced a more favorable body composition profile, reducing fat mass while preserving lean mass and thereby avoiding the muscle loss typically associated with GLP‐1 monotherapy (Figure 6E).

The GIFT NPL20 LNPs treatment exhibited both local and systemic benefits. At the injection site (iWAT), we observed a 72.2% reduction in fat mass, accompanied by significant decreases in visceral adiposity (eWAT, 36.4%) and liver weight (30.4%), with corresponding improvements in hepatic steatosis as compared to PBS group (Figure 6F). Metabolic parameters were substantially enhanced, including improved glucose tolerance, enhanced insulin sensitivity, and reduced serum TG and total TC (Figure 6G–J). Histological analysis revealed that the tri‐cistronic mRNA more effectively attenuated both adipocyte hypertrophy and hepatic lipid accumulation compared to the monotherapies (Figure 6K,L).

Quantitative PCR analysis revealed distinct advantages of the combination therapy at the molecular level. While monotherapies moderately upregulated thermogenic markers (*Ucp1* 2.7‐8.2 fold, *Pgc1α* 1.2‐2.5 fold, *Dio2* 3.2‐5.3 fold), the tri‐cistronic combination produced substantially greater increases (14.3‐, 3.8‐, and 22.5‐fold, respectively). Similarly, lipid metabolism genes showed enhanced suppression: *Fasn* and *Scd1* expression decreased from 7–51% and 17–25% with monotherapy to 63% and 85% with combination therapy, accompanied by an 3.5‐fold upregulation of *Fgf21* and significant reductions in *Pparγ* and *Leptin* (Figure 6M). These effects stem from the construct's ability to coordinate the temporal release of both hormones, leading to synergistic activation of GLP‐1 and FGF21 receptor‐mediated pathways in adipose tissue. Our findings not only validate the metabolic advantages of this dual‐hormone system but also provide crucial mechanistic insights to guide development of novel combination therapies for metabolic disorders.

## 3 Discussion and Conclusions

Protein and gene replacement therapies, such as those involving GLP‐1 agonists, FGF21, IL‐27, hold promise for treating obesity and metabolic diseases [46, 47, 48, 49]. However, recombinant protein therapies face challenges related to production stability and tissue targeting. mRNA‐based in situ protein production offers a solution by circumventing manufacturing hurdles while enabling localized expression and direct intercellular signaling modulation [50, 51]. In obesity treatment, adipose tissue is a primary target due to its central role in metabolic regulation. However, systemic administration of therapeutics is often inefficient due to limited blood perfusion in adipose depots [52, 53, 54]. Nevertheless, enhancing protein expression in adipose tissue and minimizing the metabolic impact of delivery vehicles remain critical challenges.

In this study, we developed a novel series of N‐alkyl phosphoramidate lipids (NPLs) optimized for adipose tissue delivery. By incorporating dodecyl‐modified amines, we significantly improved membrane fusion and endosomal escape, leading to an 8.6‐fold increase in transfection efficiency in iWAT. Through systematic formulation refinement, we reduced lipid content to enhance metabolic compatibility, ultimately establishing both 3CP (excluding PE or cholesterol) and 2CP (containing only NPL20 and PEG) delivery systems. Notably, removing DOPE resulted in a 5‐fold improvement in transfection efficiency compared to 4CP formulations while maintaining minimal effects on body weight and inflammation. Long‐term evaluation in high‐fat diet‐induced obese mice confirmed that repeated dosing did not induce metabolic disturbances, providing key insights for lipid excipient selection in metabolic disease applications.

The optimized NPL20‐LNP platform demonstrated high efficacy in delivering GLP‐1/FGF21 dual‐agonist mRNA to subcutaneous adipose tissue. The resulting GLP‐1/IgG4 Fc/FGF21 (GIFT) fusion protein exhibited complementary mechanisms: GLP‐1 mediated appetite suppression and improved insulin sensitivity, while FGF21 suppressed lipogenesis and promoted thermogenesis. This dual‐targeting strategy conferred substantial metabolic benefits, including reduced adiposity, amelioration of metabolic comorbidities (particularly hepatic steatosis), and preservation of lean mass—an important advantage over GLP‐1 monotherapies.

This work advances obesity treatment by establishing an mRNA delivery platform specifically tailored for adipose tissue, combined with a synergistic dual‐agonist approach. Furthermore, our systematic exploration of lipid chemistry, formulation optimization, and metabolic safety provides a framework for developing next‐generation mRNA therapies for metabolic and chronic diseases. Future integration with microneedle arrays or implantable devices may further enhance sustained delivery and translational potential. These findings not only present a promising therapeutic strategy for obesity but also establish foundational principles for mRNA‐based interventions in metabolic disorders.

## 4 Data Analysis and Statistics

All experimental data were analyzed using GraphPad Prism 9.5 software. Data are expressed as the mean ± SD as indicated in each of the figure legend. Statistical significance was determined using one‐way ANOVA followed by Dunnett's or Tukey's multiple comparisons test, or by two‐way ANOVA followed by Tukey's or Šídák's multiple comparisons test, as appropriate and indicated in the respective legends. Exact *P*‐values are provided in the figures or legends. Differences were considered statistically significant at *p* < 0.05 (^*^ *p* < 0.05, ^**^ *p* < 0.01, ^***^ *p* < 0.001, ^****^ *p* < 0.0001, unless otherwise noted).

## Author Contributions

L.M., F. X., Y.Y.H., B.M., Y.X.L. and H.J.Z. are responsible for all phases of the research. B.M., Y.X.L., H.J.Z., Y.A.F., Y.Z.X., Z.Q.J. and J.S.X. performed experiments. T.Y.Z., Y.Y.H., F. X. and L.M. provided conceptual advice and supervised the study. All the authors discussed the results and commented on the manuscript.

## Conflicts of Interest

The authors declare no conflict of interest.

## Supporting information




**Supporting File**: advs73506‐sup‐0001‐SuppMat.pdf.

## Data Availability

The data that support the findings of this study are available from the corresponding author upon reasonable request.
