# Supplementary material for: A Simplified Three‐Tailed N‐Alkyl Phosphoramidate Lipid Platform Enables Inguinal Adipose‐Accumulated mRNA Delivery for Anti‐Obesity Therapy
Source: Adv Sci (Weinh). 2025 Dec 22;13(10):e17672. doi: 10.1002/advs.202517672 (PMC12915099; doi:10.1002/advs.202517672)
Supplement: Supplementary file 1 — Supporting File: advs73506‐sup‐0001‐SuppMat.pdf. [file ADVS-13-e17672-s001.docx]

Supporting Information for

A Simplified Three-Tailed N-Alkyl Phosphoramidate Lipid Platform Enables Inguinal Adipose-Accumulated mRNA Delivery for Anti-Obesity Therapy

Bin Ma^1, 2, #^, Yunxuan Liu^1, 2, #^, Huijuan Zhang^1, 2, #^, Yian Fang^1, 2^, Yizhe Xue^1, 2^, Junsheng Xue^2^, Ziqiong Jiang^1,2^, Tianyan Zhou^2^, Yanyun Hao^1,^ ^2*^, Fei Xie^3*^, Lei Miao^1, 2,4*^

^1^State Key Laboratory of Natural and Biomimetic Drugs, School of Pharmaceutical Sciences, Peking University, Beijing, 100191 China

^2^Beijing Key Laboratory of Molecular Pharmaceutics, School of Pharmaceutical Sciences, Peking University, Beijing, 100191 China

^3^Joint Research Institute of Medical and Pharmaceutical Sciences, Qilu Hospital of Shandong University, Cheeloo College of Medicine, Shandong University, Jinan 250012, PR China.

^4^Peking University-Yunnan Baiyao International Medical Research Center, Beijing, China

#, contributing equally,

*, corresponding author, haoyanyun212@163.com, 201762000021@email.sdu.edu.cn,[[lmiao_pharm@bjmu.edu.cn](mailto:lmiao_pharm@bjmu.edu.cn)](mailto:lmiao_pharm@bjmu.edu.cn)

Contents

[Materials and Methods 3](#_Toc213770841)

[Materials 3](#_Toc213770842)

[Methods 3](#_Toc213770843)

[Cell culture and animal studies 3](#_Toc213770844)

[Metabolic Function Assessment 4](#_Toc213770845)

[RNA synthesis and expression verification 4](#_Toc213770846)

[Formulations and characterizations of LNPs 5](#_Toc213770847)

[In vivo expression of NPL LNPs 5](#_Toc213770848)

[In situ pKa determination via TNS 5](#_Toc213770849)

[Membrane fluidity experiments 6](#_Toc213770850)

[In vitro cell uptake  6](#_Toc213770851)

[Live-cell imaging of RNA release   6](#_Toc213770852)

[In vivo expression and distribution comparation 7](#_Toc213770853)

[In vivo screening of reformulated LNPs for mRNA delivery 7](#_Toc213770854)

[Cell viability 7](#_Toc213770855)

[LDH cytotoxicity assay 8](#_Toc213770856)

[LNP stability assessment 8](#_Toc213770857)

[Western blot  8](#_Toc213770858)

[Pharmacokinetics assay 9](#_Toc213770859)

[Measurement of downstream pathway activation of GIFT mRNA LNP 9](#_Toc213770860)

[Quantitative real-time PCR (qPCR) assay 9](#_Toc213770861)

[Histological and immunofluorescence staining 10](#_Toc213770862)

[The lipid alcohols and amines synthesis 10](#_Toc213770863)

[Synthetic route for the amine 11](#_Toc213770864)

[High-purity lipids synthesis 12](#_Toc213770865)

[Scheme. S1 Lipid Structures and Synthetic Methods 13](#_Toc213770866)

[Supplementary Figures 17](#_Toc213770867)

[Supplementary Tables 30](#_Toc213770868)

[Supplementary Table 1 Characterizations of PL/NPL/NPL-DLs LNPs 30](#_Toc213770869)

[Supplementary Table 2 The pKa of PL/NPL LNPs 30](#_Toc213770870)

[Supplementary Table 3 Formulation of round A orthogonal screening 31](#_Toc213770871)

[Supplementary Table 4 Characterizations of round A orthogonal screening formulations 32](#_Toc213770872)

[Supplementary Table 5 Formulation of round B screening 32](#_Toc213770873)

[Supplementary Table 6 Characterizations of round B screening formulations 32](#_Toc213770874)

[Supplementary Table 7 PK parameters of VLK and IgG4 Fc fusion proteins (calculated using NCA) 33](#_Toc213770875)

[Supplementary Table 8 List of mice primers used in qPCR 33](#_Toc213770876)

[References 33](#_Toc213770877)

**Materials and Methods**

**Materials**

2-(piperazin-1-yl)ethan-1-amine, 2-(piperidin-1-yl)ethan-1-amine, N1,N1-diethylpro pane-1,3-diamine, 3-(azepan-1-yl)propan-1-amine were purchased from Innochem (China). octadecan-1-ol, (Z)-octadec-9-en-1-ol, 2-ethylhexanoic acid, hexane-1,6-diol were purchased from Bide Pharmatech Co., Ltd. Ultra dry solvent (DCM) were purchased from J&K Scientific (China). Ionizable lipids including DLin-MC3-DMA (MC3), and 1-octylnonyl 8-(2-hydroxyethyl) 6-oxo-(undecyloxy) hexyl amino-octanoate (SM-102) were purchased from Avanti Polar Lipids, Inc. Helper lipids including cholesterol, 1,2-Dioctadecanoyl-sn-glycero-3-phophocholine (DSPC), 1,2-Dioleoyl-sn-glycero-3-phosphoethanolamine (DOPE) and 1,2-dimyristoyl-rac-glycero-3-methoxypolyethylene glycol-2000 (DMG-PEG2000) were bought from A.V.T. Pharmaceutical Tech Co., Ltd. Firefly luciferase mRNA (mFluc) was provided by Proxybio. High-fat diet (HFD) with 60% Kcal was purchased from MP Biomedicals Co.,Ltd. All the plasmid sequences were provided by Genscript Co.,Ltd. Primers was purchased from Tsingke Biotechnology Co.,Ltd. RAW-Lucia ISG (RAW ISG) was purchased from InvivoGen (France). 3T3-L1 cell lines and fetal bovine serum (FBS) were purchased from Procell Life Science & Technology Co.,Ltd. Other reagents for basal culture were bought from Meilunbio Co., Ltd. ELISA kits for mouse IL-6, IL-1β were purchased from Solarbio Science & Technology Co., Ltd. TC, TG detection kits were purchased from Jiancheng Bioengineering Research Institute (Nanjing, China).

**Methods**

**Cell culture and animal studies**

3T3-L1 cells were maintained in DMEM medium supplemented with 10% fetal bovine serum (FBS, Procell, China) and 1% penicillin/streptomycin (Meilunbio, China). RAW-Lucia ISG cells were maintained in heat inactivated DMEM medium. All cells were cultured at 37 °C in an incubator of 5% CO_2_.

All animals were purchased at Peking University Health Science Center Department of Laboratory Animal Science and all procedures were performed according to ethical regulations approved by Peking University’s Institutional Animal Care and Use Committee (Accreditation number: LA2022327). Eight-week-old male C57BL/6J mice were used to investigate the in vivo expression of LNPs. For the prophylactic model, 10-week-old male C57BL/6J mice were fed on HFD and subcutaneous administration (*s.c.*) inguinal injected with LNPs (0.67 mg/kg) weekly. For the therapeutic models, 8-week-old male C57BL/6J mice were induced obesity after 13 weeks of HFD feeding and then *s.c.* inguinal injected with LNPs (0.75 mg/kg) twice per week for another 5 weeks. Body weight was monitored twice per week, and body composition was determined by EchoMRI. Glucose tolerance test (GTT) and insulin tolerance test (ITT)were conducted during the treatment. The mice were enthusiastic for tissues and serum collection. Serum triglycerides (TG), total cholesterol (TC) were measured according to the manufacturer’s protocol.

**Metabolic Function Assessment**

For the GTT, mice were fasted for 12 h and then received an intraperitoneal (*i.p.*) injection of 20% glucose solution (prepared in deionized water) at 2 g/kg body weight. Blood glucose was measured using ACCU-CHEK Active Blood Glucose Meter (Roche) at the indicated time points (0, 15 min, 30 min, 60 min, 120 min). For the ITT, mice were fasted for 4 h and then *i.p.* injected with insulin (0.75 U/kg body weight, prepared in 0.9% saline). Blood glucose was measured at the indicated time points (0 min, 15 min, 30 min, 60 min, 120 min). Blood samples for detection were collected via tail vein bleeding. The first droplet was discarded and the second droplet was used for subsequent analysis.

**RNA synthesis and expression verification**

mGLP-1/VLK Fc/FGF21, mGLP-1/IgG4 Fc/FGF21(GIFT), mGLP-1/IgG4 Fc, mIgG4 Fc/FGF21 and mFluc were synthesized using T7 polymerase mediated in vitro transcription (IVT) system from linearized pUC57 plasmid vectors containing T7 promoter, 5′ and 3′ untranslated regions (UTRs) and a poly A tail (100 nt). The clean-cap AG 5′ capping (Cap 1) and 1-methylpsuedo-uridine UTP were added to the transcription reaction. The uridine-5′triphosphate (UTP) was fully replaced with 1-methylpsudeo-uridine UTP. IVT reactions were conducted according to the manufacturer’s protocols (Novoprotein, China). Expression verification of mGLP-1/VLK Fc/FGF21, mGIFT, mGLP-1/IgG4 Fc, IgG4 Fc/FGF21 were visualized using western blot analysis.

**Formulations and characterizations of LNPs**

LNPs were formulated using the microfluidic mixing method^[1]^. In brief, calculated ionizable lipids, cholesterol, phospholipid, and DMG-PEG2000 were dissolved in ethanol. mRNA was diluted in sodium citrate buffer (pH 4.0). The ethanol phase and the aqueous phase were rapidly mixed using a microfluidic chip device at a 1:3 ethanol/water volume ratio. LNPs were further dialyzed overnight using pH 7.4 PBS at 4℃. The particle size, PDI and zeta potential of LNPs were measured by dynamic light scattering (Zetasizer Nano ZSP, Malvern). mRNA concentration and encapsulation efficiency (EE) of LNP were measured using Quant-it RiboGreen RNA assay (Invitrogen).

**In vivo expression of NPL LNPs**

For construction of PL/NPL/NPL-DLs LNPs, different PL/NPL/NPL-DLs lipids/DOPE/cholesterol/ DMG-PEG2000 molar ratio was set as 35/16/46.5/2.5. MC3, SM-102 LNPs were set as the positive control. MC3 /SM-102 LNPs were formulated with an ionizable lipids/DSPC/cholesterol/DMG-PEG2000 molar ratio of 50/10/38.5/1.5. The mice received *s.c.* into the inguinal white adipose tissue (iWAT) region of encapsulating mFluc RNA LNPs. At 6 h post injection, mice were intraperitoneally injected with 150 µL d-luciferin (potassium salt) (20 mg/mL in 1xPBS), and bioluminescence imaging was performed using IVIS imaging system (Perkin Elmer).

**In situ pKa determination via TNS**

The apparent pKa values of individual PL/NPL lipids were determined using the fluorescent probe 2-(p-toluidino)-6-naphthalenesulfonic acid (TNS) within preformed LNPs. These LNPs were composed of PL/NPL lipid/DOPE/cholesterol/DMG-PEG2000 at a molar ratio of 35:16:46.5:2.5, dispersed in 1×PBS at a total lipid concentration of ~6 mM. Briefly, a 100 μM stock solution of TNS was prepared in distilled water. LNPs were diluted to a total lipid concentration of 100 μM in 90 μL of buffered solutions (prepared in triplicate). These buffers contained 10 mM HEPES, 10 mM 4-morpholineethanesulfonic acid, 10 mM ammonium acetate, and 130 mM NaCl, with pH values ranging from 2.71 to 11.5. 10 μL TNS stock solution were added to each LNP-buffer mixture in a black 96-well plate and thoroughly mixed. Fluorescence intensity was measured using a Tecan Pro200 plate reader at excitation and emission wavelengths of 321 nm and 445 nm, respectively. The resulting fluorescence data were plotted as a sigmoidal curve of fluorescence intensity versus buffer pH. The apparent pKa of the LNP formulation was determined as the logarithm of the inflection point of this curve.

**Membrane fluidity experiments**

The membrane fluidity of PL/NPL LNPs was measured based on the fluorescence anisotropy of 7-methylcoumarin (*λ*_ex_ = 315 nm, *λ*_em_ = 386 nm) from fluorescence polarization (lifetime and steady state spectrometer FLS980)^[2]^. 7-Methylcoumarin (10 μL, 100 μM) in DMSO was added into LNP (0.2 mL). The following equation was used to calculate the polarity (*P*):

*P*=(*I*∥−*I*⊥)/(*I*∥+*I*⊥)

Membrane fluidity was expressed as 1/*P* since polarity is inversely proportional to fluidity.

**In vitro cell uptake**

3T3-L1 cells were plated in 96-well plates at a density of ~10^4^ cells per well 24 h prior to the experiment. DiR containing LNP (DiR-LNP) encapsulating mRNA were added to each well at a concentration gradient (0.1µg, 0.2 and 0.4 µg mRNA per well). For the DiR-LNP, DiR was added to LNP as a five component, the PL/NPL lipids/DOPE/Cholesterol/DMG-PEG2000/Dir molar ratio of 38.5/16/46.5/2.5/1 was used. After 2 h of incubation at 37 °C, the wells were washed 2 times with cold PBS and replaced with fresh media. Cellular uptake of LNPs was then assessed by flow cytometry, with gating strategy shown in **Supplementary Fig. 16.**

**Live-cell imaging of RNA release**

Intracellular release of RNA from LNPs was visualized using an inverted Leica STELLARIS 8 confocal microscope. Encapsulating Cy5-labeled RNA were applied for tracing the intracellular behavior of RNA escape from endosome. 3T3-L1 cells were treated with the LNPs with labeled mRNA at a concentration of 1 μg/mL and incubated for 3 h at 37 °C. Subsequently, the media were replaced with fresh media containing 1 μg/mL Hoechst 33342 and lysotracker (FITC), and endosome release was observed using a 60× Plan-Neofluar 1.3 numerical aperture (NA) oil-immersion objective. For all experiments, the field of view (FOV) was set to 354, 25 μm × 354, 25 μm (full). The pinhole was set to 1 Airy Unit for all channels. The acquired imaging data were processed using LAS X Office and ImgeJ for analysis.

**In vivo expression and distribution comparation**

DiR-LNP encapsulating mFluc RNA was *s.c.* into the iWAT region of mice (0.15 mg/kg mRNA). At 6 h post injection, fluorescence and bioluminescence imaging of the tissues of mice were performed using IVIS imaging system. For the separation of adipocytes and non-adipocytes, iWAT was minced and digested in 20 mg/mL BSA in HBSS containing collagenase I (2 mg/ml). Digestion was performed at 37 ℃ for 20 min with a vortex of every three minutes, and then centrifuged at 700 × g for 5 min at 4 ℃. The upper (adipocyte-enriched) and precipitate pellet (non-adipocyte) layers were separated and homogenized prior to detections. Fluorescence was measured by Firefly-Glo Luciferase Reporter Assay Kit (Yeasen Biotechnology Co., Ltd.) following the manufacturer’s protocol, using BioTek synergy H1 microplate reader. The fluorescence image of DiR was visualized by Typhoon.

**In vivo screening of reformulated LNPs for mRNA delivery**

For the round A screening, an orthogonal array table L16 (4)^4^ was used to optimize the LNP formulations (Supplementary Table 3). For the round B screening, different ratios of NPL20/PEG2000 were used (Supplementary Table 5). Calculated NPL20, cholesterol, DOPE and DMG-PEG2000 were dissolved in ethanol as the ethanol phase, and then was used to formulate different LNPs. The mice received *s.c.* of encapsulating mFluc RNA LNPs (0.15 mg/kg mRNA) into the inguinal white adipose tissue (iWAT) region. At 6 h post injection, bioluminescence imaging was performed using IVIS imaging system.

**Cell viability**

A density of ~2×10^4^ 3T3-L1 cells per well were seeded into 96-well plate and incubated for 24 h at 37 ℃, 5% CO_2_. The medium was replaced by LNPs containing medium at a concentration of 100 ng mRNA per well (100 μL). After 48 h incubation, add 10 μL of MTT solution (5 mg/mL) to each well, and incubate in the incubator for 4 hours. Discard the residual liquid and add 100 μL of DMSO to each well and for 10 minutes. Absorbance was measured at 570 nm wavelength using BioTek synergy H1 microplate reader. Cell viability was expressed as percentage in respect to untreated cells.

**LDH cytotoxicity assay**

LDH cytotoxicity assay was carried out following a previously mentioned method^[3]^. In brief, a density of ~2×10^4^ 3T3-L1 cells per well were seeded into 96-well plate and incubated for 24 h at 37℃,5% CO_2_. The medium was replaced by LNPs containing medium at serials concentrations of mRNA per well. After 24 h incubation, cytotoxicity was measured using the LDH cytotoxicity assay kit (C0017, Beyotime) according to manufacturer’s instructions.

**LNP stability assessment**

mLuc-encapsulating LNPs (4CP, 3CP-w/o PE, 3CP-w/o CHO, 2CP, and SM-102) were prepared as mentioned above. For accelerated stability studies, LNPs were stored at 4°C. For long-term stability, LNPs were cryopreserved at –80°C after adding an equal volume of sucrose solution (87 mg/mL) as a cryoprotectant. Particle size, encapsulation efficiency, and zeta potential were measured at day 7, 14, 21, and 28. To evaluate transfection efficiency at different time points, 3T3-L1 cells seeded in 96-well plates were treated with LNPs (100 ng/well). After 6 h, luciferase expression was quantified using a Luciferase Reporter Assay Kit (Yeasen Biotechnology) according to the manufacturer's protocol and the expression levels were normalized to values measured at day 0.

**Western blot**

For testing the expression of target proteins mediated by LNPs delivering GLP-1/VLK Fc/FGF21, GIFT, GLP-1/IgG4 Fc, IgG4 Fc/FGF21 mRNA, 3T3-L1 cells were plated in 6-well plates at a density of ~2.5×10^5^ cells per well 12 h prior to the experiment. After treated by the LNPs for 24 h, Cells lysates were extracted using RIPA lysis buffer (Solarbio) supplemented with PMSF protease inhibitor. The BCA protein assay kit (Solarbio) was used to determine protein concentrations. Proteins were separated on 10% gel sodium dodecyl sulfate–polyacrylamide gel electrophoresis and transferred onto polyvinylidene fluoride. Membranes were blocked 1 h and then incubated overnight at 4℃ with primary antibodies: HA tag (Abcam, #ab3724, 1:1000) and GAPDH (CST, #5174S, 1:1000). Membranes were washed with TBST three times and then incubated with secondary HRP-conjugated antibodies for 1 h at room temperature. Unbound antibodies were washed with TBST three times. Chemiluminescence imaging was applied for protein band visualization.

**Pharmacokinetics assay**

The HFD mice were s.c. treated with NPL20 LNPs containing mGLP-1/IgG4 Fc/FGF21 (with HA-Tag) or mGLP-1/VLK/FGF21 (with HA-Tag) in the inguinal region. The iWAT (adjacent to injection site) and blood samples were harvested at 0.25, 0.5, 1, 2, 3, 5 and 7 days post injection. The level of the fusion protein in the samples was quantitated using a HA-Tag ELISA kit (MEIMIAN) following the manufacturer’s protocol. The pharmacokinetic (PK) parameters for each subject were calculated using noncompartmental analysis (NCA) with the PKNCA package (version 0.12.1) in R (version 4.5.1). The analysis was performed for VLK and IgG4 Fc fusion proteins separately, using the concentration-time data obtained from iWAT and serum. Key NCA parameters included the maximum observed concentration (C_max_), the area under the concentration-time curve (AUC) from time zero to the last measurable time point (AUC_0-t_). The mean and standard deviation (SD) for each of these PK parameters were then calculated for VLK and IgG4 Fc fusion proteins in iWAT and serum.

**Measurement of downstream pathway activation of GIFT mRNA LNP**

To assess the activation of downstream signaling pathway mediated by GLP-1, tissues (iWAT) cAMP levels were measured using a cAMP ELISA kit (BioVendor) according to the manufacturer’s instructions. Furthermore, to confirm FGF21-mediated pathway activation, phosphorylation of ERK and AKT was assessed in 3T3-L1 cells by Western blot. Briefly, cells seeded in 12-well plates were treated with NPL20 mGIFT LNP (100 ng/mL) for 24 h, followed by immunoblotting using the following antibodies: p-ERK1/2 (Abmart, #T40072), ERK1/2 (Abmart, #T40071), p-AKT (Abmart, #T40067), and AKT (Abmart, #T55561).

**Quantitative real-time PCR (qPCR) assay**

Total RNA was isolated from iWAT tissue using the TransZol Up Plus RNA Kit (TransGen Biotech), followed by cDNA synthesis with HiScript III All-in-one RT SuperMix (Vazyme). Quantitative PCR was performed using SYBR Green-based qPCR SuperMix (TransGen Biotech) on a QuantStudio™ 6 Flex system (v1.7.1 software). B2M served as the reference gene, and relative quantification was calculated using the 2^-(ΔΔCt) method. Primer sequences are provided in **Supplementary Table 8**.

**Histological and immunofluorescence staining**

The liver and iWAT of mice were fixed in 4% PFA and embedded in paraffin. Hematoxylin and eosin (H&E) staining were conducted by Wuhan Servicebio Technology Co., Ltd, and the images were taken on tissue paraffin WS-10 scanner with NDP view 2 (Zhiyue Medical Technology, Hefei) and quantified by Image J 1.53u. The quantification of adipocytes areas in iWAT sections was analyzed using ImageJ (Adiposoft Plugin).

For immunofluorescence, paraffin sections of iWAT were stained using Multi-target detection kit (Wellgene, #RD1401) according to the manufacturer’s instructions. Adipocyte cell membrane and macrophage were stained using Caveolin-1 (Abcam, #192869, 1:300 dilution) and F4/80 (CST, #70076T, 1:500 dilution) antibodies, and the corresponding dyes were WG570 (Ex 555 nm, Em 570 nm) and WG520 (Ex 488 nm, Em 519 nm), respectively. The expression of mFluc was determined using Firefly luciferase antibody (Abcam, #185924, 1:500 dilution) and dye WG650 (Ex 633 nm, Em 650 nm). Nucleic was visualized using fluorescent mounting medium with DAPI (ZSGB, #ZLI-9600). The images were scanned using Vectra Polaris and processed using ImageJ.

**The lipid alcohols and amines synthesis**

The lipid alcohols R_1_a, R_1_b and amine A1-A4 were purchased from Bide Pharmatech Co., Ltd and Innochem (China) company (**Scheme. S1**), another lipid alcohol B7 was obtained via esterification. Amino A1-12C to A4-12C underwent bromination to yield their corresponding brominated derivatives. The structure was confirmed by ^1^H NMR spectrometry (Bruker AVANCE-400 NMR spectrometer with a Magnex Scientific superconducting magnet).

**Synthetic route for the lipid alcohol B7**

To a stirred solution of hexane-1,6-diol (2.36 g, 20 mmol) and 2-ethylhexanoic acid (1.44 g, 10 mmol) in DCM (35 mL) was added DCC (2.47 g, 12 mmol) and DMAP (147 mg, 1.2 mmol). The reaction mixture was stirred at rt for 16 h, then went through vacuum suction filtration, and the filtrate was added with water. The layers were separated and the aqueous layer was extracted three times with DCM. The combined organic phases were washed with saturated citric acid aqueous solution, then dried Na_2_SO_4_ and concentrated. The product was purified by column chromatography on silica, eluted with petroleum ether/ethyl acetate (10:1), to give ester B7 (1.63 g, 67.3 %) as a colourless oil. **^1^H NMR** (400 MHz, CDCl_3_) δ 4.09 (t, *J*=8Hz, 2H), 3.65 (t, *J*=8Hz, 2H), 2.29-2.22 (m, 1H), 1.76-1.56 (m, 8H), 1.50-1.37 (m, 4H), 1.33-1.22 (m, 4H), 0.89 (t, *J*=8Hz, 6H).

**Synthetic route for the amine**

To a stirred solution of amine (A1-A4, 10 mmol) and 1-bromododecane (1.98g, 8 mmol) in DMF (20 mL) was added K_2_CO_3_ (1.38 g, 10 mmol). The reaction mixture was stirred at rt for 12 h, then water (60 mL) was added. The aqueous layer was extracted three times with dichloromethane. The combined organic phases were washed with saturated sodium chloride, then dried Na_2_SO_4_and concentrated. The product was purified by column chromatography, eluted with DCM/ MeOH/ 34% NH_3_·H_2_O (45:5:1) to obtain amine (A1-12C, A2-12C, A3-12C, A4-12C) as a colourless oil.

**A1-12C ^1^H NMR** (400 MHz, CDCl_3_) δ 3.46-3.42 (m, 4H), 2.73 (t, *J*=8Hz, 2H), 2.63 (t, *J*=8Hz, 2H), 2.52 (t, *J*=8Hz, 2H), 2.44-2.39 (m, 4H), 1.79-1.70 (m, 2H), 1.53-1.46 (m, 2H), 1.31-1.28 (m, 16H), 0.90 (t, *J*=8Hz, 3H).

**A2-12C ^1^H NMR** (400 MHz, CDCl_3_) δ 2.72 (t, *J*=8Hz, 2H), 2.62 (t, *J*=8Hz, 2H), 2.47-2.37 (m, 6H), 1.61-1.55 (m, 4H), 1.51-1.44 (m, 4H), 1.33-1.26 (m, 18H), 0.89 (t, *J*=8Hz, 3H).

**A3-12C** **^1^H NMR** (400 MHz, CDCl_3_) δ 2.65 (t, *J*=8Hz, 2H), 2.60 (t, *J*=8Hz, 2H), 2.55-2.46 (m, 6H), 1.70-1.63 (m, 2H), 1.53-1.43 (m, 2H), 1.37-1.26 (m, 18H), 1.02 (t, *J*=8Hz, 6H), 0.88 (t, *J*=8Hz, 3H).

**A4-12C** **^1^H NMR** (400 MHz, CDCl_3_) δ 2.74 (t, *J*=8Hz, 2H), 2.52 (t, *J*=8Hz, 2H), 2.46-2.32(m, 6H), 1.72-1.44 (m, 12H), 1.39-1.26 (m, 18H), 0.88 (t, *J*=8Hz, 9H).

**High-purity lipids synthesis**

**Scheme. S1 Lipid Structures and Synthetic Methods**

The lipids from PL1 to NPL28 are synthesized using the same methods. To a stirred solution of POCl_3_ (166 mg, 1 mmol) in DCM (10 mL) was added a DCM solution of R_1_-OH (2 mmol) with Et_3_N (202 mg, 2 mmol). The reaction mixture was stirred at rt for 3 h, then the mixture solution was added slowly dropwise to the DCM solution of Amine A-NH_2_ or A-NH_2_-R_2_ (1 mmol) and Et_3_N (101 mg, 1 mmol), and then stirred at rt for 3h. After completion of the reaction, wash with saturated sodium chloride, and concentrate under reduced pressure to obtain the crude product. The product was purified by column chromatography, eluted with DCM: MeOH (15:1) to obtain the compound. All lipid structures are confirmed by ^1^H NMR spectrometry (Bruker AVANCE-400 NMR spectrometer with a Magnex Scientific superconducting magnet). The selected lipid compound PL16, PL17, NPL19 and NPL20 was ultimately characterized and confirmed by ^1^H NMR**,** ^13^C NMR (Bruker AVANCE-400 NMR spectrometer with a Magnex Scientific superconducting magnet), and mass spectrometry (Waters Xevo G2 QTOF and ThermoFisher Orbitrap Exploris 240) **(Supplementary Fig. 1-12)**.

**PL1** yield:47.6%. Rf:0.53. **^1^H NMR** (400 MHz, CDCl_3_) δ 4.01-3.81 (m, 4H), 3.19-2.92 (m, 6H), 2.63-2.44 (m, 6H), 1.70-1.59 (m, 4H), 1.36-1.28 (m, 60H), 0.90 (t, *J*=8Hz, 6H).

**PL2** yield:42.5%. Rf:0.56.**^1^H NMR** (400 MHz, CDCl_3_) δ 5.4-5.32 (m, 4H), 4.00-3.90 (m, 4H), 3.23-3.11 (m, 6H), 2.76-2.52 (m, 6H), 2.05-2.00 (m, 4H), 1.68-1.65 (m, 4H), 1.41-1.27 (m, 44H), 0.90 (t, *J*=8Hz, 6H).

**PL4** yield:46.1%. Rf:0.53. **^1^H NMR** (400 MHz, CDCl_3_) δ 4.09 (t, *J*=8Hz, 4H), 4.01-3.91 (m, 4H), 3.21-3.03 (m, 6H), 2.68-2.47 (m, 6H), 2.30-2.23 (m, 2H), 1.70-1.23 (m, 32H), 0.90 (t, *J*=8Hz, 12H).

**PL5** yield:77.2%. Rf:0.67. **^1^H NMR** (400 MHz, CDCl_3_) δ 5.39-5.31 (m, 4H), 4.03-3.92 (m, 4H), 3.09-3.02 (m, 2H), 2.56-2.51 (m, 6H), 2.04-1.99 (m, 8H), 1.99-1.59 (m, 8H), 1.49-1.26 (m, 46H), 0.89 (t, *J*=8Hz, 6H).

**PL6** yield:69.4%. Rf:0.68. **^1^H NMR** (400 MHz, CDCl_3_) δ 4.09 (t, *J*=8Hz, 4H), 4.03-3.99 (m, 4H), 3.14-3.11 (m, 2H), 2.78-2.62 (m, 6H), 2.30-2.25 (m, 2H), 2.16-1.81 (m, 8H), 1.69-1.25 (m, 36H), 0.90 (t, *J*=8Hz, 12H).

**PL7** yield:78.5%. Rf:0.61. **^1^H NMR** (400 MHz, CDCl_3_) δ 4.03-3.95 (m, 4H), 3.04-2.98 (m, 2H), 2.48-2.43 (m, 6H), 1.70-1.58 (m, 8H), 1.48-1.27 (m, 62H), 0.90 (t, *J*=8Hz, 6H).

**NPL8** yield:65.2%. Rf:0.61. **^1^H NMR** (400 MHz, CDCl_3_) δ 3.98-3.86 (m, 4H), 3.31-3.13 (m, 2H), 3.04-2.94 (m, 2H), 2.72-2.39 (m, 6H), 1.70-1.58 (m, 8H), 1.56-1.40 (m, 4H), 1.38-1.24 (m, 78H), 0.89 (t, *J*=8Hz, 9H).

**NPL9** yield:62.8%. Rf:0.73. **^1^H NMR** (400 MHz, CDCl_3_) δ 5.41-5.32 (m, 4H), 3.99-3.89 (m, 4H), 3.32-3.11 (m, 2H), 3.01-2.95 (m, 2H), 2.69-2.35 (m, 6H), 2.05-2.01 (m, 8H), 1.70-1.61 (m, 8H), 1.55-1.48 (m, 4H), 1.49-1.28 (m, 62H), 0.90 (t, *J*=8Hz, 9H).

**NPL10** yield:71.8%. Rf:0.70. **^1^H NMR** (400 MHz, CDCl_3_) δ 4.11-4.07 (m, 4H), 3.98-3.86 (m, 4H), 3.31-3.14 (m, 2H), 3.00-2.93 (m, 2H), 2.70-2.32 (m, 6H), 2.29-2.24 (m, 2H), 1.69-1.27 (m, 58H), 0.90 (t, *J*=8Hz, 15H).

**NPL11** yield:42.5%. Rf:0.68. **^1^H NMR** (400 MHz, CDCl_3_) δ 4.03-3.89 (m, 4H), 3.24-3.15 (m, 4H), 2.05-2.92 (m, 4H), 2.63-2.44 (m, 6H), 1.70-1.61 (m, 4H), 1.36-1.28 (m, 80H), 0.90 (t, *J*=8Hz, 9H)

**NPL12** yield:48.3%. Rf:0.71. **^1^H NMR** (400 MHz, CDCl_3_) δ 5.38-5.31(m, 4H), 4.00-3.75 (m, 4H), 3.24-2.59 (m, 14H), 2.05 -2.01 (m, 8H), 1.68-1.28 (m, 64H), 0.90 (t, *J*=8Hz, 9H).

**NPL13** yield:45.1%. Rf:0.79. **^1^H NMR** (400 MHz, CDCl_3_) δ 4.09 (t, *J*=8Hz, 4H), 4.01-3.89 (m, 4H), 3.26-2.62 (m, 14H), 2.30 -2.23 (m, 2H), 1.48-1.23 (m, 52H), 0.90 (t, *J*=8Hz, 9H).

**PL15** yield:87.6%. Rf:0.53. **^1^H NMR** (400 MHz, CDCl_3_) δ 4.01-3.93 (m, 4H), 3.06-2.99 (m, 2H), 2.69 -2.64 (m, 6H), 1.76-1.62 (m, 6H), 1.38-1.22 (m, 64H), 1.12 (t, J= 8Hz, 6H), 0.88 (t, *J*=8Hz, 6H).

**PL16** yield:82.3%. Rf:0.74. **^1^H NMR** (400 MHz, CDCl_3_) δ 5.38-5.29 (m, 4H), 3.99-3.91 (m, 4H), 3.04-2.98 (m, 2H), 2.71 -2.65 (m, 6H), 2.03-1.98 (m, 8H), 1.78-1.71 (m, 2H),1.66-1,61 (m, 4H), 1.35-1.25 (m, 44H), 1.20 (t, *J*=8Hz, 6H). 0.87 (t, *J*=8Hz, 6H). **^13^C NMR** (101 MHz, CDCl_3_) δ 129.94, 129.74, 66.34, 66.28, 50.85, 46.61, 40.25, 31.88, 30.44, 30.37, 29.74, 29.50, 29.45, 29.29, 29.23, 29.20, 27.21, 27.19, 27.18, 25.60, 22.65, 14.08, 10.57. MS (ESI) m/z [M] ^+^ calculated: 710.6454, found: 711.6490.

**PL17** yield:88.1%. Rf:0.66. **^1^H NMR** (400 MHz, CDCl_3_) δ 4.08 (t, *J*=8Hz, 4H), 4.01-3.96 (m, 4H), 3.12-3.04 (m, 2H), 2.88 -2.74 (m, 6H), 2.30-2.23 (m, 2H), 1.89-1.79 (m, 2H), 1.71-1.21 (m, 38H), 0.90 (t, *J*=8Hz, 6H). **^13^C NMR** (101 MHz, CDCl_3_) δ 176.48, 66.24, 66.18, 63.87, 53.42, 50.47, 47.36, 46.57, 39.81, 31.77, 30.35, 30.28, 29.61, 28.60, 26.87, 25.58, 25.47, 25.26, 22.60, 13.93, 11.83, 10.06. MS (ESI) m/z [M] ^+^ calculated: 662.4999, found: 663.5057.

**NPL18** yield:77.3%. Rf:0.67. **^1^H NMR** (400 MHz, CDCl_3_) δ 4.03-3.83 (m, 4H), 3.10-2.68 (m, 8H), 1.89-1.77 (m, 2H), 1.69-1.48 (m, 6H), 1.35-1.16 (m, 86H), 0.88 (t, *J*=8Hz, 9H).

**NPL19** yield:77.7%. Rf:0.69. **^1^H NMR** (400 MHz, CDCl_3_) δ 5.40-5.32 (m, 4H), 4.00-3.83 (m, 4H), 3.13-2.74 (m, 8H), 2.05-1.91 (m, 10H), 1.68-1.49 (m, 6H), 1.38-1.23 (m, 70H), 0.90 (t, *J*=8Hz, 9H). **^13^C NMR** (101 MHz, CDCl3) δ 129.97, 129.73, 66.26, 66.20, 49.88, 46.65, 45.87, 45.84, 43.61, 43.56, 31.92, 31.90, 30.44, 30.37, 29.76, 29.70, 29.68, 29.65, 29.62, 29.59, 29.52, 29.49, 29.45, 29.43, 29.36, 29.31, 29.26, 29.23, 28.51, 28.49, 27.21, 26.88, 25.66, 22.67, 14.10. MS (ESI) m/z [M] ^+^ calculated: 878.8332, found: 879.8376.

**NPL20** yield:85.4%. Rf:0.64. **^1^H NMR** (400 MHz, CDCl_3_) δ 4.09 (t, *J*=8Hz, 4H), 3.98-3.88 (m, 4H), 3.20-2.91 (m, 8H), 2.30-2.23 (m, 2H), 2.15-2.08 (m, 2H), 1.99-1.79 (m, 6H), 1.69-1.23 (m, 54H), 0.90 (t, *J*=8Hz, 15H). **^13^C NMR** (101 MHz, CDCl_3_) δ 176.35, 176.32, 66.19, 66.13, 63.74, 63.68, 49.37, 47.26, 46.50, 46.41, 45.91, 45.88, 43.19, 43.14, 31.81, 31.69, 30.25, 30.18, 30.06, 29.99, 29.57, 29.53, 29.50, 29.34, 29.25, 28.52, 28.34, 28.32, 26.77, 25.49, 25.44, 25.40, 25.20, 25.09, 24.98, 23.25, 22.58, 22.51, 14.02, 13.86, 11.76, 8.56, 8.53. MS (ESI) m/z [M] ^+^ calculated.: 830.6877, found: 831.6940.

**NPL-DL21** yield:79.1%. Rf:0.61. **^1^H NMR** (400 MHz, CDCl_3_) δ 4.09 (t, *J*=8Hz, 4H), 3.99-3.83 (m, 4H), 3.19-2.92 (m, 8H), 2.29-2.23 (m, 2H), 2.11-2.05 (m, 2H), 1.70-1.23 (m, 44H), 0.96-0.88 (m, 15H).

**NPL-DL22** yield:64.1%. Rf:0.64.**^1^H NMR** (400 MHz, CDCl_3_) δ 4.09 (t, *J*=8Hz, 6H), 3.99-3.83 (m, 4H), 3.09-2.85 (m, 8H), 2.30-2.23 (m, 3H), 1.90-1.21 (m, 56H), 0.90 (t, *J*=8Hz, 18H).

**PL23** yield:74.6%. Rf:0.66. **^1^H NMR** (400 MHz, CDCl_3_) δ 4.02-3.88 (m, 4H), 3.07-2.75 (m, 4H), 1.79-1.63 (m, 14H), 1.37-1.27 (m, 64H), 0.90 (t, *J*=8Hz, 6H).

**PL24** yield:78.2%. Rf:0.59. **^1^H NMR** (400 MHz, CDCl_3_) δ 5.41-5.32 (m, 4H), 4.01-3.96 (m, 4H), 3.61-2.89 (m, 8H), 2.18-1.83 (m, 16H), 1.70-1.63 (m, 6H), 1.36-1.27 (m, 44H), 0.90 (t, *J*=8Hz, 6H).

**PL25** yield:71.9%. Rf:0.69. **^1^H NMR** (400 MHz, CDCl_3_) δ 4.09 (t, *J*=8Hz, 4H), 4.02-3.97 (m, 4H), 3.60-2.90 (m, 8H), 2.30-2.08 (m, 6H), 1.88-1.83 (m, 2H), 1.70-1.23 (m, 36H), 0.90 (t, *J*=8Hz, 12H).

**NPL26** yield:67.5%. Rf:0.59. **^1^H NMR** (400 MHz, CDCl_3_) δ 3.99-3.85 (m, 4H), 3.47-2.80 (m, 8H), 2.06-1.49 (m, 14H), 1.39-1.28 (m, 80H), 0.90 (t, *J*=8Hz, 9H).

**NPL27** yield:72.3%. Rf:0.63. **^1^H NMR** (400 MHz, CDCl_3_) δ 5.41-5.32 (m, 4H), 3.99-3.86 (m, 4H), 3.57-2.90 (m, 8H), 2.18-1.83 (m, 16H), 1.68-1.59 (m, 6H), 1.51-1.28 (m, 64H), 0.90 (t, *J*=8Hz, 9H).

**NPL28** yield:72.4%. Rf:0.69. **^1^H NMR** (400 MHz, CDCl_3_) δ 4.09 (t, *J*=8Hz, 4H), 4.02-3.88 (m, 4H), 3.15-2.90 (m, 8H), 2.23-1.23 (m, 64H), 0.90 (t, *J*=8Hz, 15H).

**Supplementary Figures**


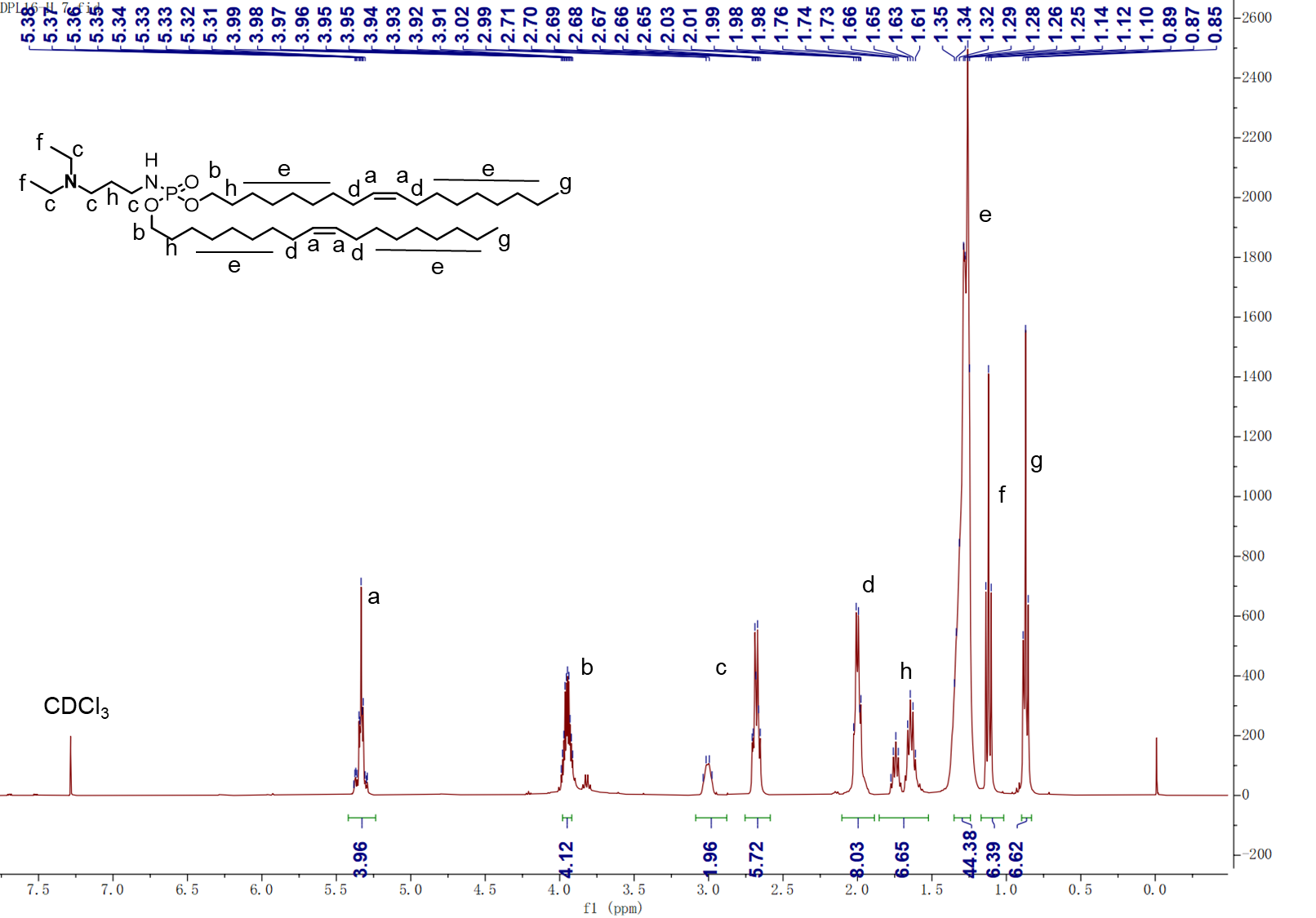


**Supplementary Fig. 1. ^1^H NMR spectrum (400 MHz, CDCl_3_, 298 K) of compound PL16.**


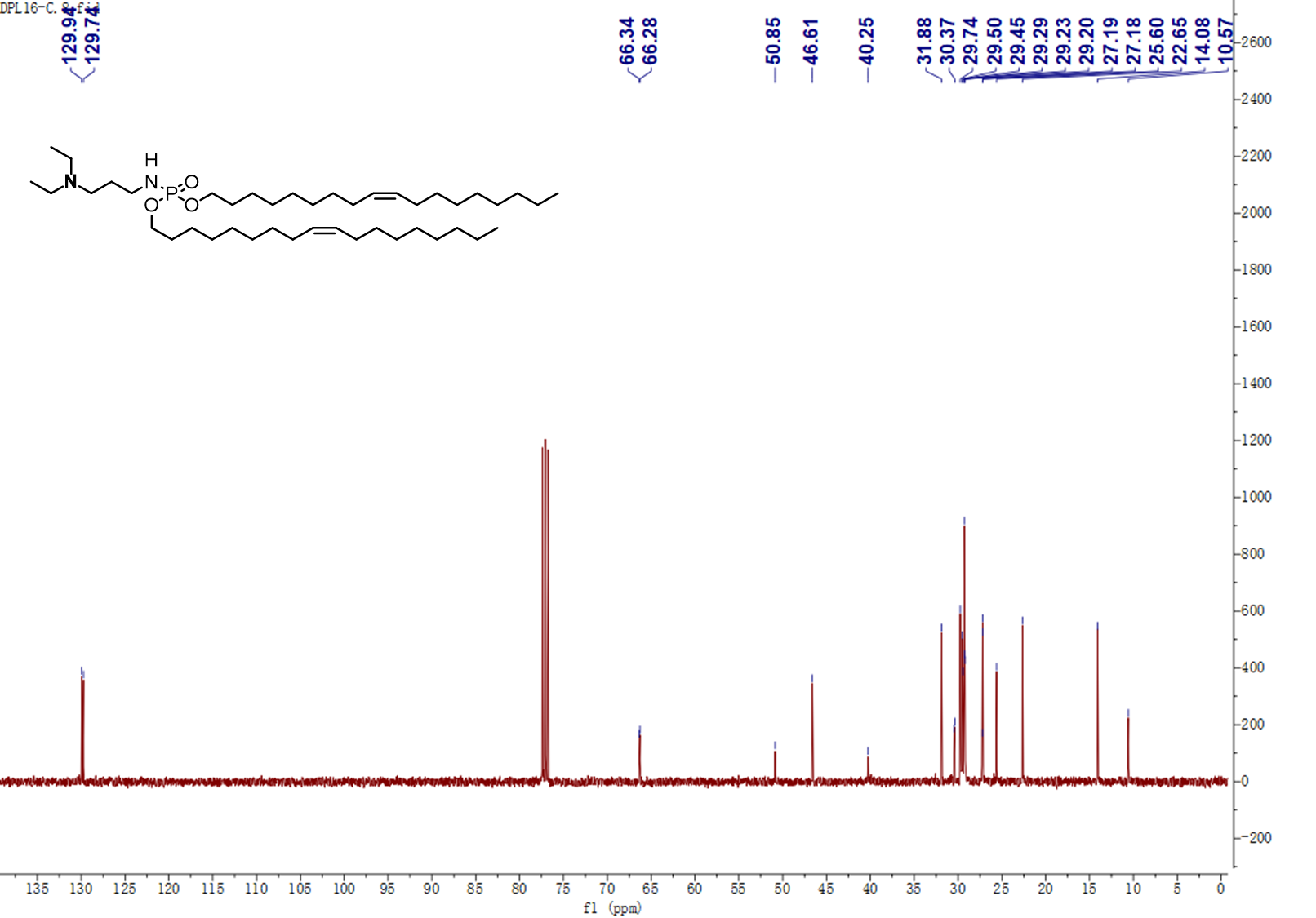


**Supplementary Fig. 2. ^13^C NMR spectrum (400 MHz, CDCl_3_, 298 K) of compound PL16.**

**Supplementary Fig. 3. MS spectrum of compound PL16.**


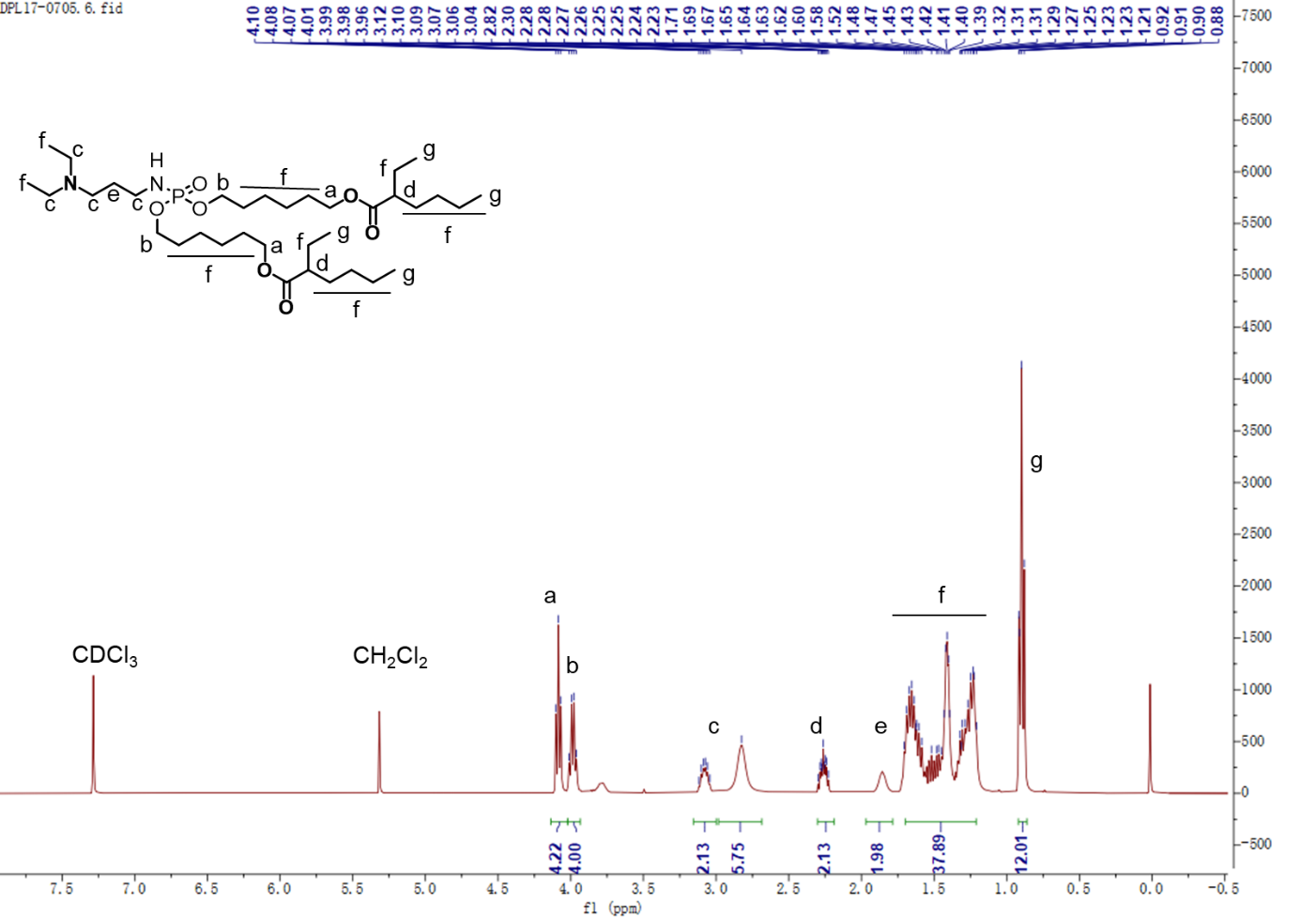


**Supplementary Fig. 4. ^1^H NMR spectrum (400 MHz, CDCl_3_, 298 K) of compound PL17.**


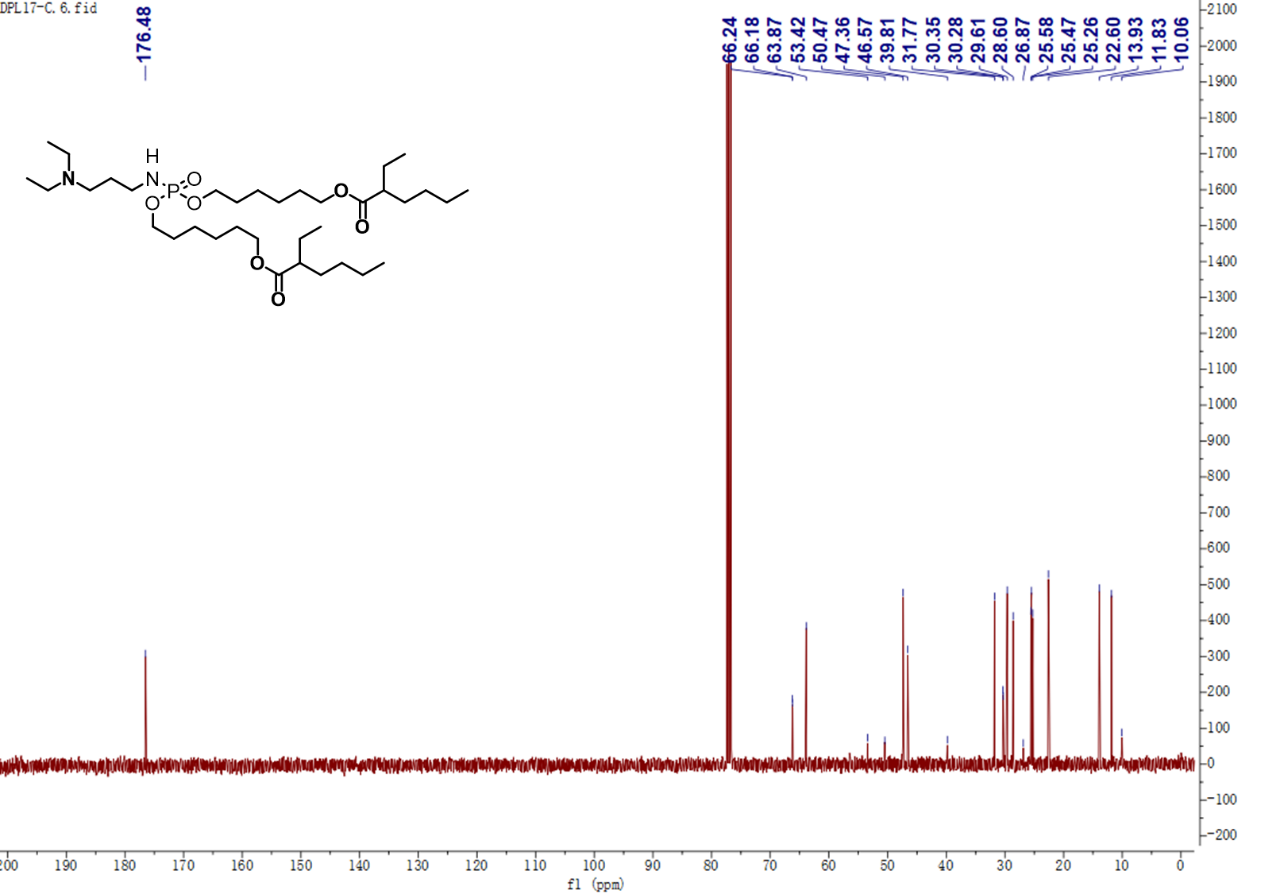


**Supplementary Fig. 5. ^13^C NMR spectrum (400 MHz, CDCl_3_, 298 K) of compound PL17.**

**Supplementary Fig. 6. MS spectrum of compound PL17.**


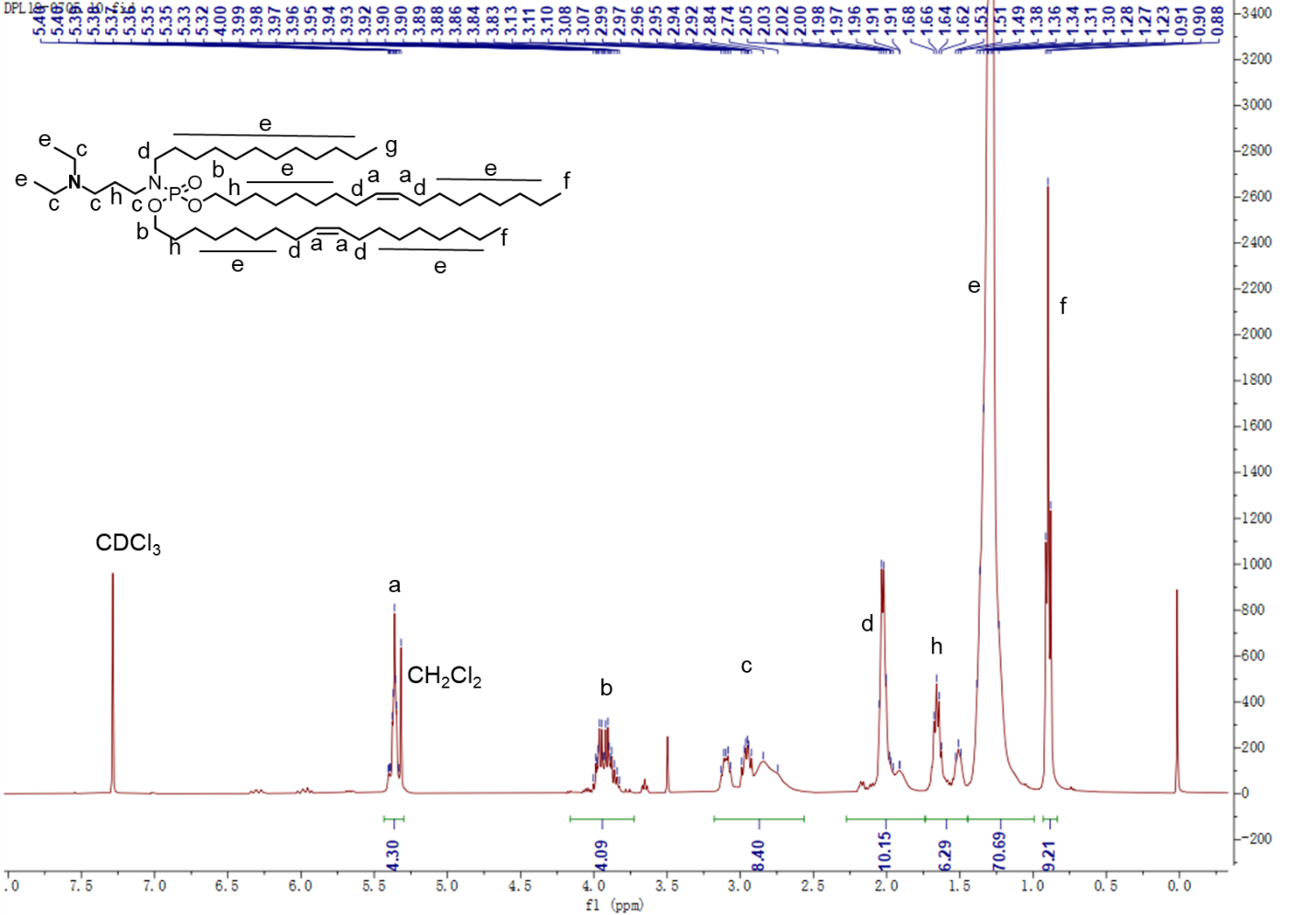


**Supplementary Fig. 7. ^1^H NMR spectrum (400 MHz, CDCl_3_, 298 K) of compound NPL19.**


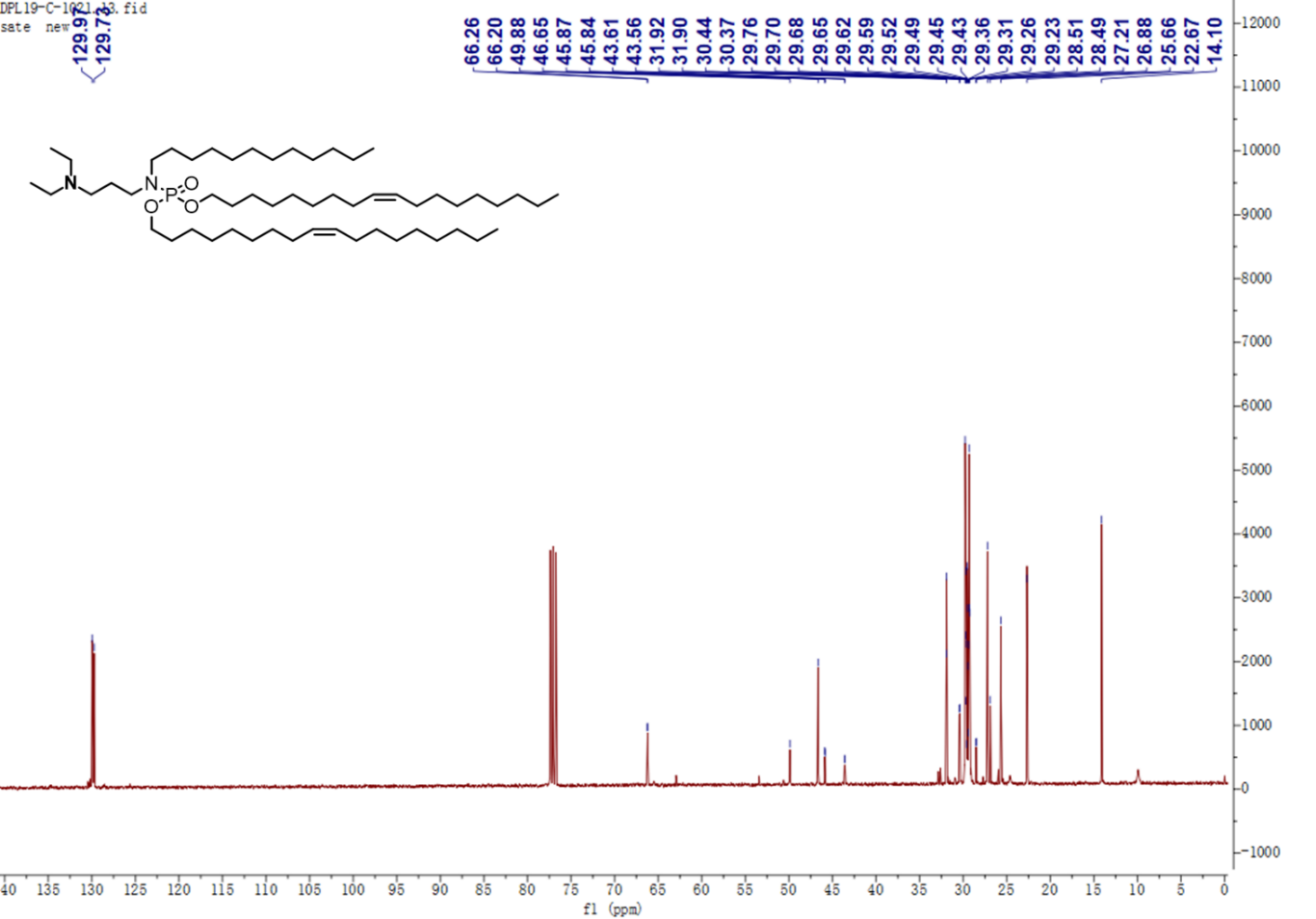


**Supplementary Fig. 8. ^13^C NMR spectrum (400 MHz, CDCl_3_, 298 K) of compound NPL19.**

**Supplementary Fig. 9. MS spectrum of compound NPL19.**


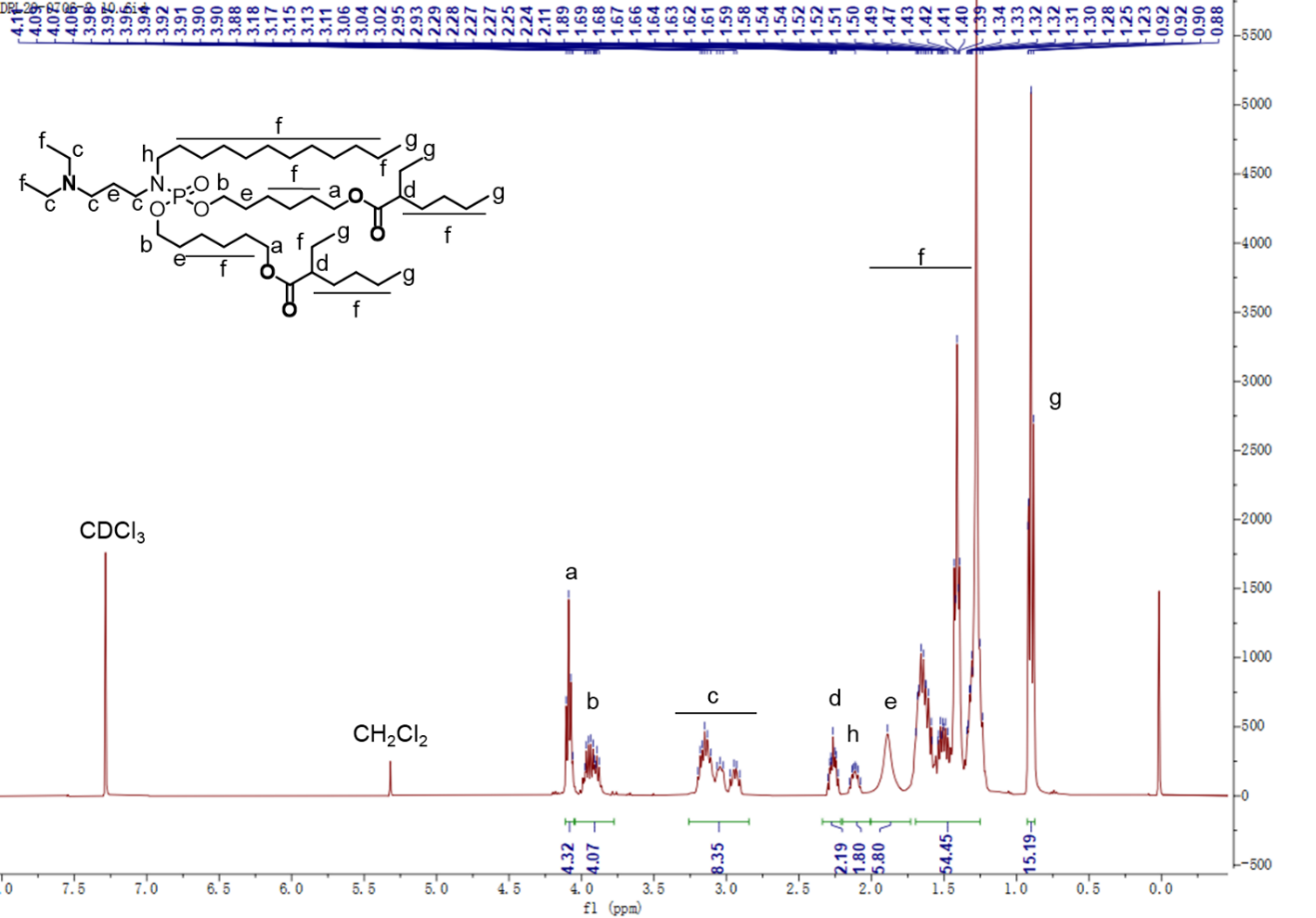


**Supplementary Fig. 10. ^1^H NMR spectrum (400 MHz, CDCl_3_, 298 K) of compound NPL20.**


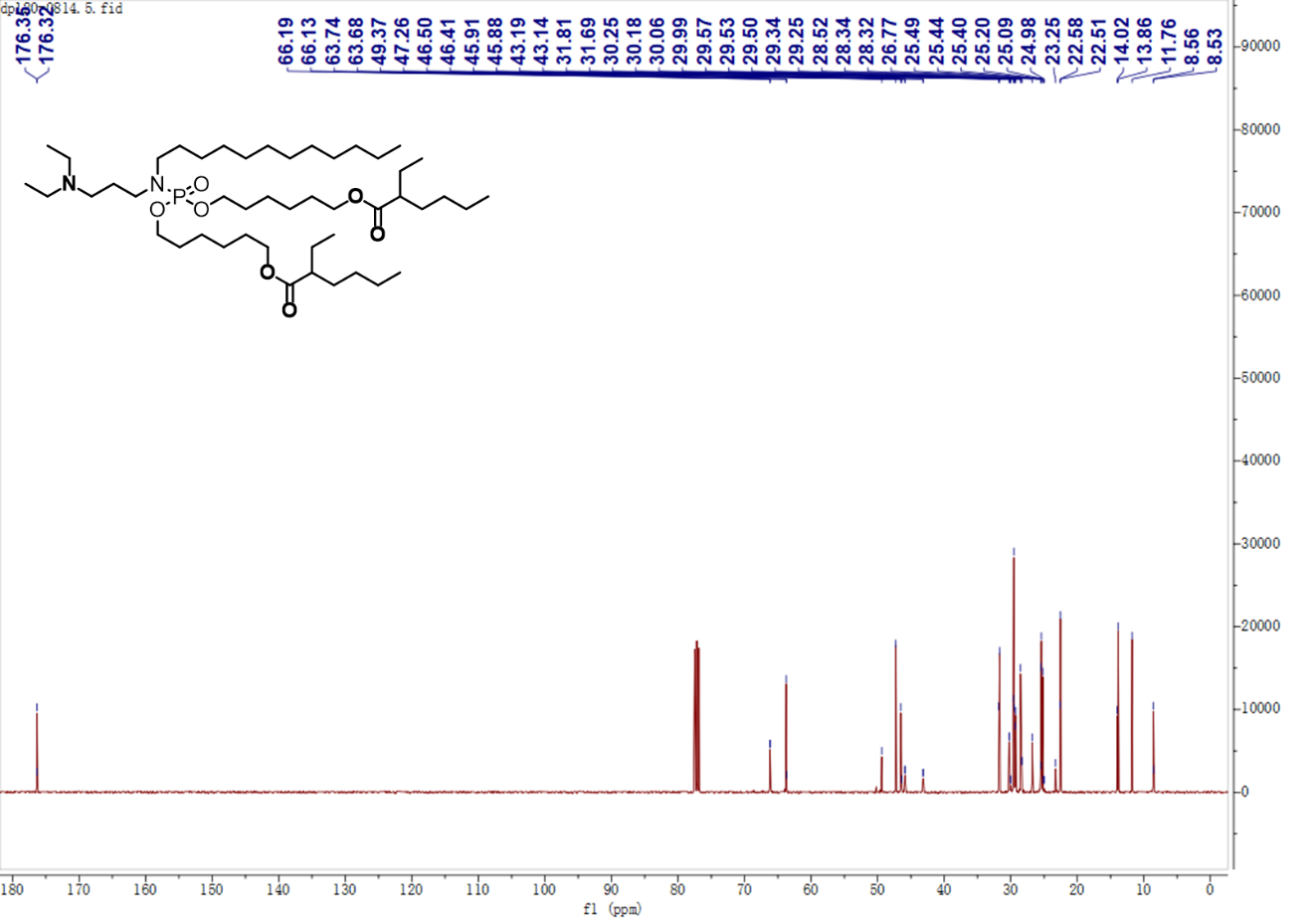


**Supplementary Fig. 11. ^13^C NMR spectrum (400 MHz, CDCl_3_, 298 K) of compound NPL20.**

**Supplementary Fig. 12. MS spectrum of compound NPL20.**


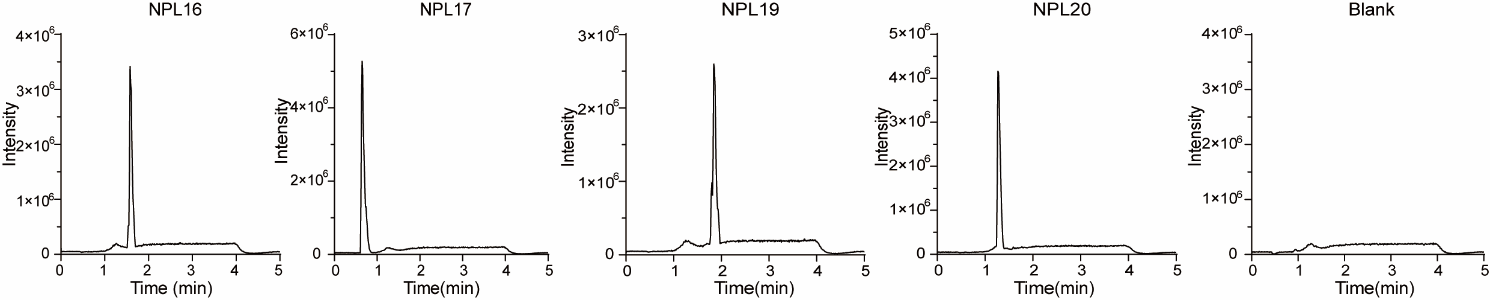


**Supplementary Fig. 13. LC-MS spectrum of compound PL16, PL17, NPL19 and NPL20.**


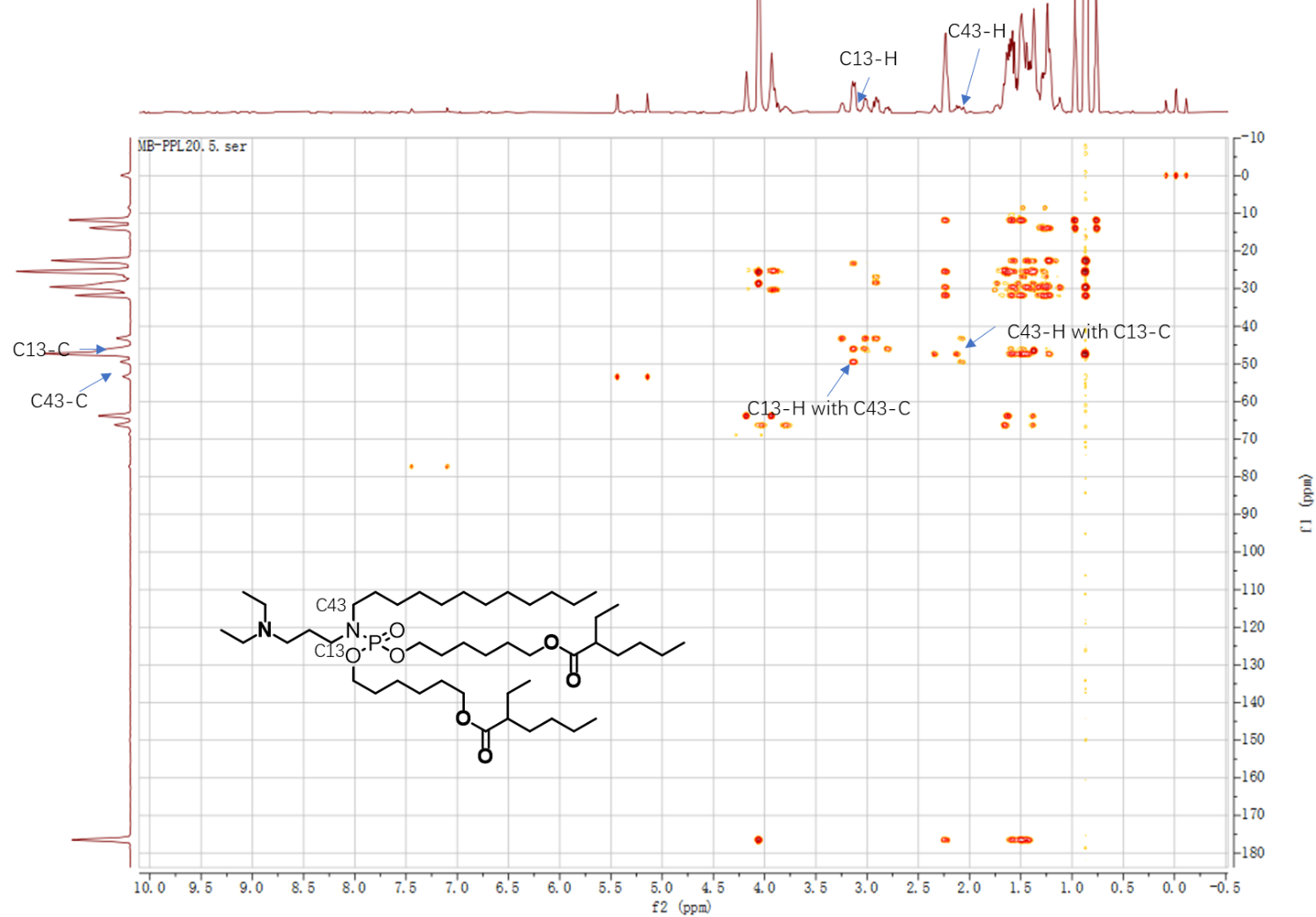


**Supplementary Fig. 14. HMBC spectrum of compound NPL20.**

**Supplementary Fig. 15. Structural characteristics of lipid PL16, PL17, NPL19 and NPL20 analyzed using Avogadro software.** (A) Schematic diagram of key bond angle configurations (created with BioRender.com), and quantitative analysis results of bond lengths (Å) and bond angles (°). (B) Three-dimensional spatial conformations of PL16, PL17, NPL19, and NPL20 constructed using Avogadro molecular modeling software, rendered with high-precision ball-and-stick composite models.


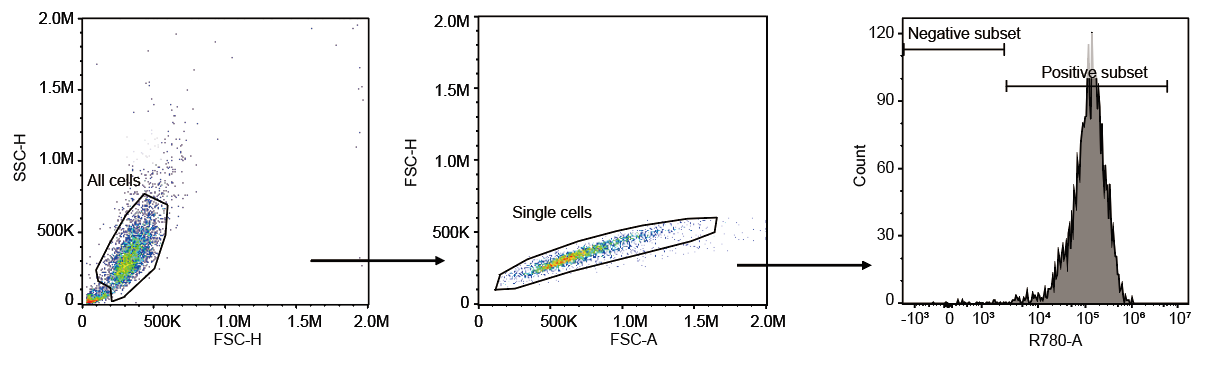


**Supplementary Fig. 16. Flow cytometry gating strategy.**


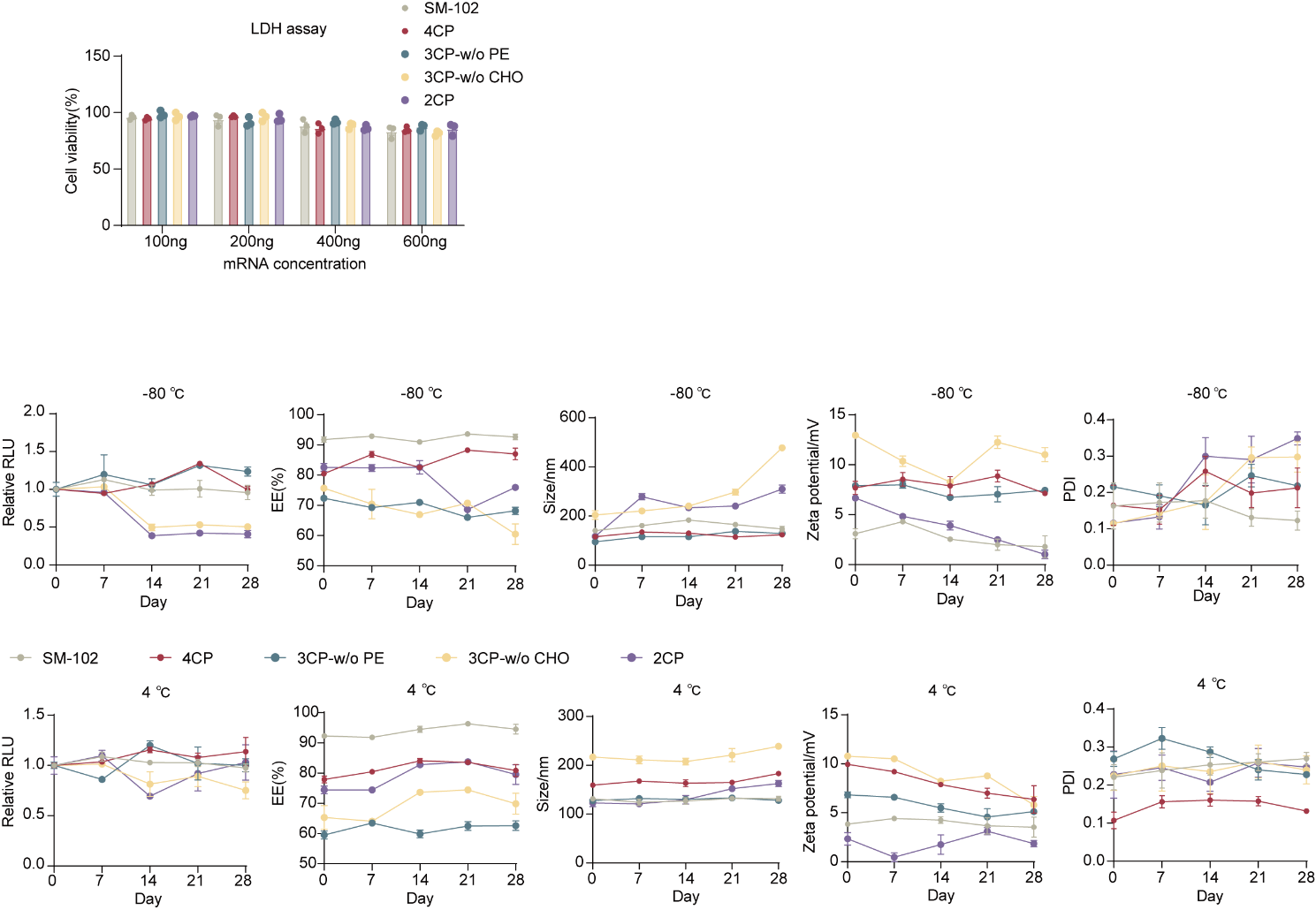


**Supplementary Fig. 17. LNP stability under different storage conditions.** Evaluation of mFluc expression, encapsulation efficiency (EE%), size, PDI, and zeta potential after 28 days of storage at 4°C and -80°C (n=3), all data presented as mean ± SEM.


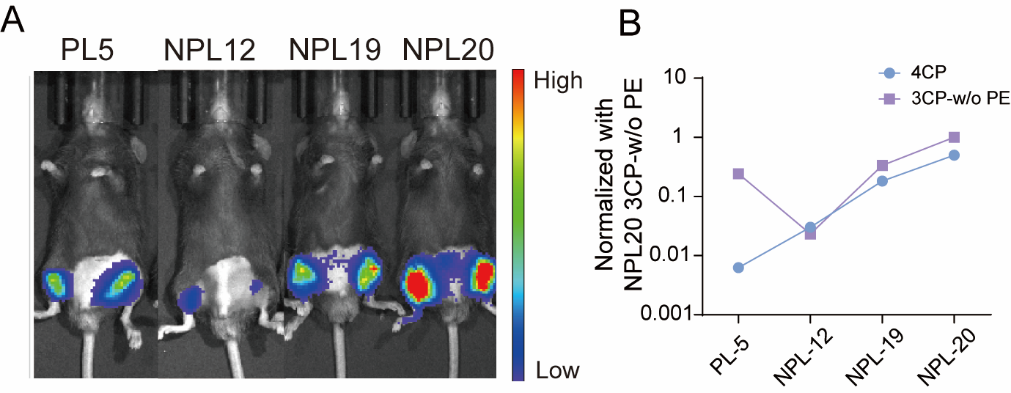


**Supplementary Fig. 18. Evaluation of the lead 3CP-PE formulation for universal expression enhancement.** (A) In vivo bioluminescence imaging of mFluc expression 6 h after bilateral s.c. administration of NPL LNPs (0.25 mg/kg) into the iWAT of C57BL/6J mice. (B) Comparison of expression between the 4CP and 3CP-w/o PE formulations, with all data normalized to the NPL20 3CP-w/o PE group, data presented as mean value.


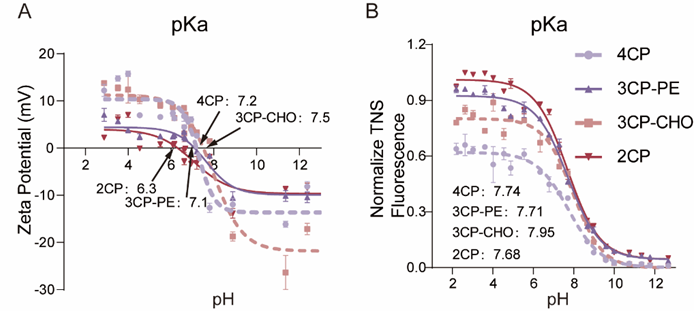


**Supplementary Fig. 19. Characterization of candidate LNP formulations.** (A) Zeta potential and (B) apparent pKa determined by the TNS fluorescence-based assay. Data are shown from n=2 independent measurements.


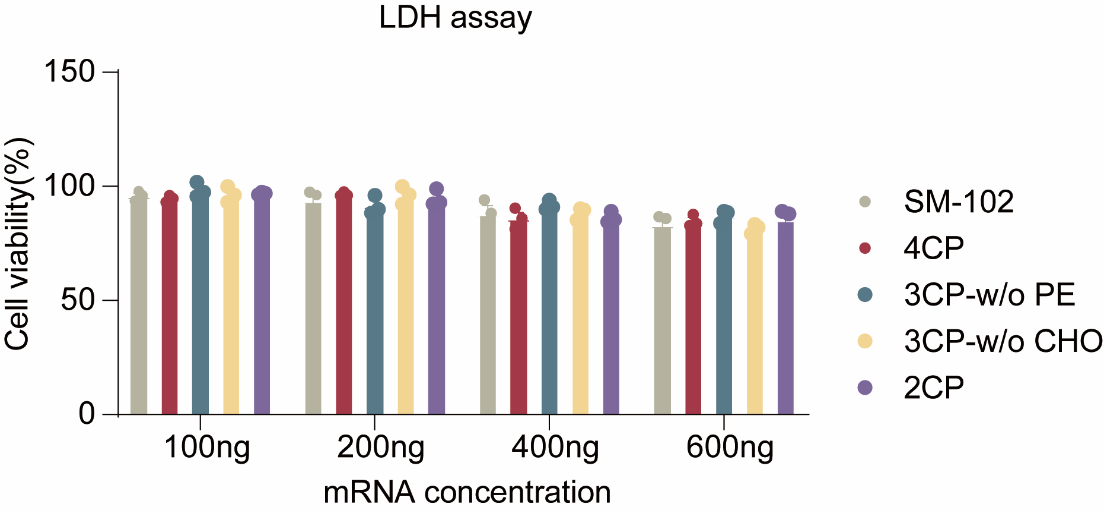


**Supplementary Fig. 20. The cytotoxicity of NPL20 LNPs against 3T3-L1 cells was assessed by the LDH assay across a mRNA concentration range of 100 to 600 ng/well (n=3).** All data presented as mean ± SEM.


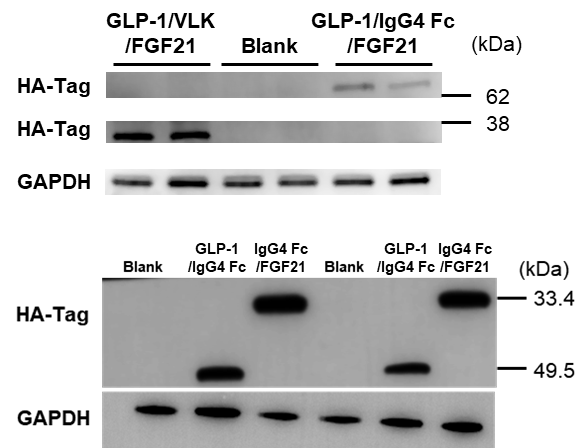


**Supplementary Fig. 21. Expression of GLP-1/VLK/FGF21, GLP-1/IgG4 Fc/FGF21, GLP-1/IgG4 Fc, IgG4 Fc/FGF21 mRNA.** Expression of protein in 3T3-L1 cells pretreated with NPL20 LNPs (1µg/6 well plate) for 24 h (n=2 independent tests, the experiments were repeated for additional twice with similar results).


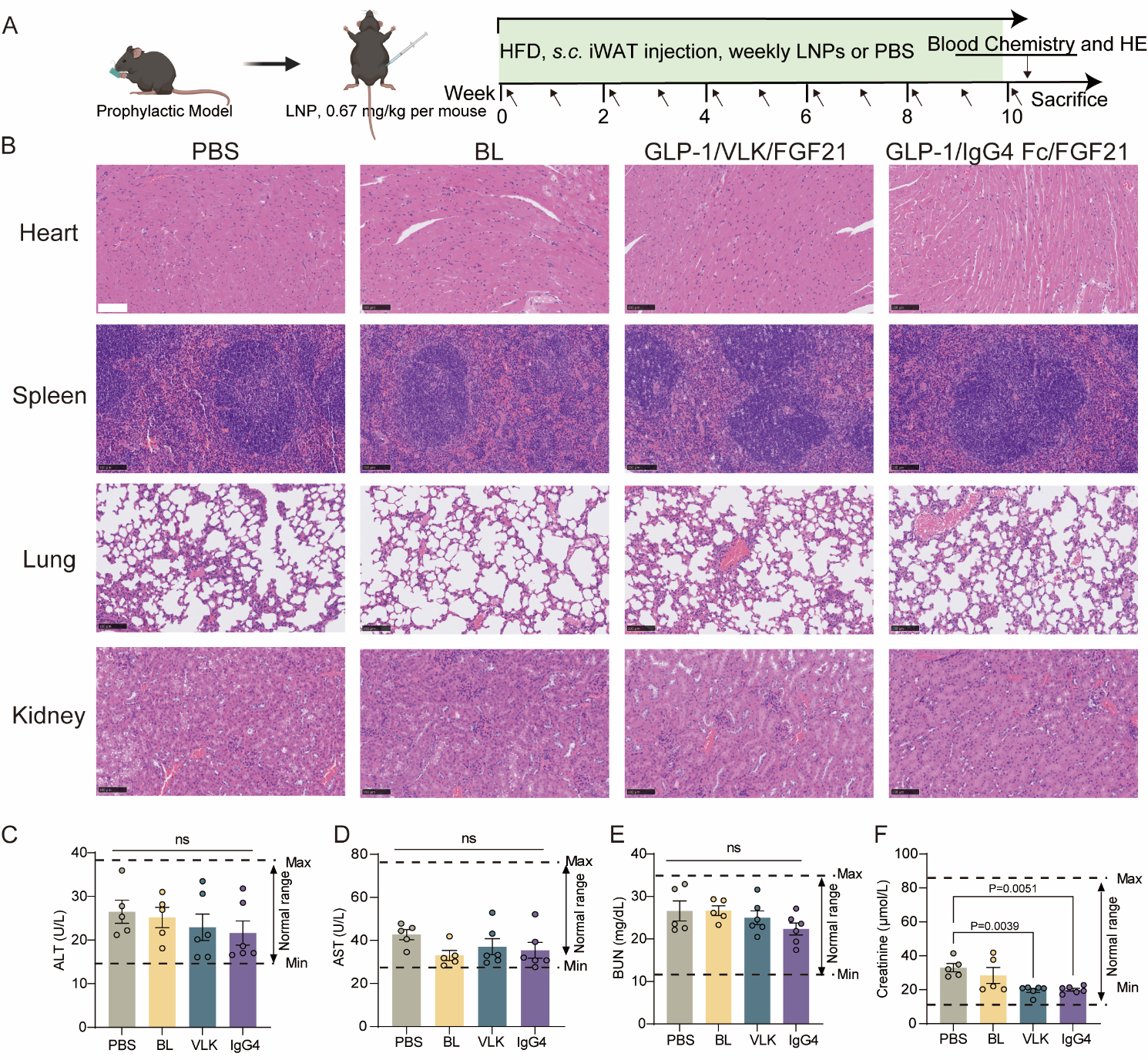


**Supplementary Fig. 22. Safety assessment of GIFT mRNA NPL20 LNPs.** A) Schematic of the experimental design for prophylactic model. Male mice were subcutaneously injected (s.c.) in the inguinal white adipose tissue (iWAT) region with NPL20 LNPs without/with mRNA (6.7 mg/kg NPL20 BL LNPs, equivalent to 0.67 mg/kg NPL20 mRNA LNPs) or PBS weekly since the beginning of HFD feeding. B) In a preventive study, mice were sacrificed after ten-week administrations, and their hearts, spleens, lungs, and kidneys were collected for histological analysis. Representative images (from one independent experiment, n=5-6 mice per group) showed no obvious pathological alterations in any of the major organs. Scale bar represents 100 µm. C-F) To evaluate hepatic and renal function, blood was collected from the mice prior to sacrifice for biochemical analysis. Serum levels of aspartate aminotransferase (AST), alanine aminotransferase (ALT), blood urea nitrogen (BUN), and creatinine (CREA) all remain within normal ranges. Data are presented as mean ± SEM (n=5-6). Statistics were calculated using One-way ANOVA followed by Dunnett's multiple comparisons test using GraphPad Prism software (version 9.5).

**Supplementary Tables**

**Supplementary Table 1 Characterizations of PL/NPL/NPL-DLs LNPs**

| **Name** | **Diameter (nm)** | **PDI** | **Zeta potential(mV)** | **EE of mRNA%** |
| --- | --- | --- | --- | --- |
| PL1 | 222.3±6.8 | 0.38±0.01 | 10.27±0.59 | 0 |
| PL2 | 155.1±1.8 | 0.36±0.01 | 12.60±0.92 | 53.7 |
| PL4 | 122.4±2.8 | 0.28±0 | 4.74±0.27 | 72.6 |
| PL5 | 193.0±2.9 | 0.20±0.03 | 16.20±1.68 | 57.1 |
| PL6 | 110.4±1.7 | 0.21±0.02 | 4.16±1.57 | 60.9 |
| PL7 | 224.1±14.6 | 0.59±0.03 | 17.20 ±0.44 | 68.0 |
| PL15 | 160.1±9.8 | 0.39±0.08 | 13.63±1.88 | 79.0 |
| PL16 | 131.4±1.2 | 0.29±0.04 | 11.67±1.23 | 81.3 |
| PL17 | 76.6±1.1 | 0.24±0.01 | 3.35±0.89 | 59.8 |
| PL23 | 254.1±16.0 | 0.35±0.01 | 13.60±1.35 | 92.3 |
| PL24 | 170.4±4.2 | 0.23±0.04 | 10.43±0.61 | 77.7 |
| PL25 | 192.0±3.8 | 0.28±0.02 | 0.42±0.66 | 49.9 |
| NPL8 | 191.0±7.9 | 0.34±0.05 | 13.97±0.68 | 35.0 |
| NPL9 | 123.1±1.2 | 0.12±0.01 | -0.46±1.29 | 73.0 |
| NPL10 | 94.1±0.6 | 0.13±0.03 | 4.00±0.65 | 83.3 |
| NPL11 | >1000 | 0.84±0.06 | -9.91±1.64 | 0 |
| NPL12 | 108.7±1.9 | 0.23±0.03 | 6.57±0.34 | 65.9 |
| NPL13 | 120.3±1.2 | 0.33±0.05 | 3.52±0.83 | 58.8 |
| NPL18 | 129.7±2.7 | 0.29±0.03 | 0.52±0.72 | 31.1 |
| NPL19 | 117.1±4.2 | 0.35±0.01 | 13.20±0.75 | 70.0 |
| NPL20 | 85.1±2.0 | 0.17±0.02 | 9.85±0.35 | 84.6 |
| NPL DL21 | 193.9±1.0 | 0.12±0.01 | 9.93±0.31 | 78.7 |
| NPL DL22 | 148.9±3.8 | 0.14±0.04 | 8.88±1.16 | 86.2 |
| NPL26 | 159.6±3.7 | 0.25±0.01 | 13.60±1.61 | 83.5 |
| NPL27 | 283.8±4.8 | 0.29±0.01 | 10.43±2.05 | 63.1 |
| NPL28 | 165.4±2.3 | 0.24±0.02 | 10.63±0.38 | 70.7 |

**Supplementary Table 2 The pKa of PL/NPL LNPs**

| Name | PL16 | PL17 | NPL19 | NPL20 |
| --- | --- | --- | --- | --- |
| pKa | 9.42 | 9.05 | 7.92 | 7.78 |

**Supplementary Table 3 Formulation of round A orthogonal screening**

| **Name** | **Factors (molar ratio)** | | | | **NPL20/mRNA**  **wt.ratio** | **N/P** | **Liposome/**  **mRNA wt. ratio** |
| --- | --- | --- | --- | --- | --- | --- | --- |
|  | **NPL20** | **DOPE** | **Cholesterol** | **DMG-PEG_2000_** |  |  |  |
| A1 | 15 | 0 | 0 | 0 | 10 | 11.9 | 10.0 |
| A2 | 15 | 10 | 15 | 1 | 10 | 11.9 | 22.6 |
| A3 | 15 | 20 | 25 | 2.5 | 10 | 11.9 | 34.7 |
| A4 | 15 | 30 | 35 | 5 | 10 | 11.9 | 48.8 |
| A5 | 25 | 0 | 15 | 2.5 | 10 | 11.9 | 15.8 |
| A6 | 25 | 10 | 0 | 5 | 10 | 11.9 | 19.6 |
| A7 | 25 | 20 | 35 | 0 | 10 | 11.9 | 23.7 |
| A8 | 25 | 30 | 25 | 1 | 10 | 11.9 | 26.6 |
| A9 | 35 | 0 | 25 | 5 | 10 | 11.9 | 17.6 |
| A10 | 35 | 10 | 35 | 2.5 | 10 | 11.9 | 19.4 |
| A11 | 35 | 20 | 0 | 1 | 10 | 11.9 | 16.0 |
| A12 | 35 | 30 | 15 | 0 | 10 | 11.9 | 19.7 |
| A13 | 45 | 0 | 35 | 1 | 10 | 11.9 | 14.3 |
| A14 | 45 | 10 | 25 | 0 | 10 | 11.9 | 14.6 |
| A15 | 45 | 20 | 15 | 5 | 10 | 11.9 | 18.9 |
| A16 | 45 | 30 | 0 | 2.5 | 10 | 11.9 | 17.6 |
| 4CP | 35 | 16 | 46.5 | 2.5 | 10 | 11.9 | 22.4 |

**Supplementary Table 4 Characterizations of round A orthogonal screening formulations**

| **Name** | **Diameter (nm)** | **PDI** | **Zeta potential(mV)** | **EE of mRNA%** |
| --- | --- | --- | --- | --- |
| A1 | >1000 | 0.95±0.10 | -4.95±1.27 | 0 |
| A2 | 168.5±3.3 | 0.14±0.02 | 11.93±0.75 | 67.3 |
| A3 | 104.1±2.6 | 0.20±0.03 | 5.22±1.17 | 78.4 |
| A4 | 123.5±7.2 | 0.26±0.01 | 4.67±0.60 | 57.3 |
| A5 | 117.8±2.1 | 0.27±0.03 | 5.48±0.93 | 57.7 |
| A6 | 136.1±7.4 | 0.26±0.01 | 1.00±0.40 | 40.8 |
| A7 | >1000 | 0.83±0.22 | 0.27±1.23 | 0 |
| A8 | 180.9±1.0 | 0.13±0.03 | 12.50±0.50 | 67.9 |
| A9 | 139.4±12.5 | 0.44±0.14 | 3.91±0.60 | 55.4 |
| A10 | 124.4±1.8 | 0.17±0.03 | 7.00±0.80 | 75.6 |
| A11 | 211.2±4.8 | 0.18±0.04 | 10.13±4.11 | 63.3 |
| A12 | >1000 | 0.56±0.39 | -0.98±0.63 | 0 |
| A13 | 169.8±5.6 | 0.24±0.04 | 10.93±0.31 | 80.9 |
| A14 | >1000 | 0.61±0.30 | -3.76±2.34 | 0 |
| A15 | 101.3±0.2 | 0.17±0.05 | 4.82±0.31 | 56.0 |
| A16 | 226.3±6.4 | 0.17±0.01 | 11.57±1.29 | 60.9 |
| 4CP | 136.4±7.7 | 0.10±0.01 | 7.90±0.51 | 80.2 |

**Supplementary Table 5 Formulation of round B screening**

| **Name** | **Factors (molar ratio)** | | **NPL20/mRNA wt.ratio** | **N/P** | **Liposome/mRNA wt.ratio** |
| --- | --- | --- | --- | --- | --- |
|  | **NPL20** | **DMG-PEG_2000_** |  |  |  |
| B1 | 25 | 2.5 | 10 | 11.9 | 13.0 |
| B2 | 35 | 2.5 | 10 | 11.9 | 12.2 |
| B3 | 50 | 2.5 | 10 | 11.9 | 11.5 |
| B4 | 75 | 2.5 | 10 | 11.9 | 11.0 |

**Supplementary Table 6 Characterizations of round B screening formulations**

| **Name** | **Diameter (nm)** | **PDI** | **Zeta potential(mV)** | **EE of mRNA%** |
| --- | --- | --- | --- | --- |
| B1 | 118.8±2.6 | 0.25±0.05 | 2.31±0.86 | 72.4 |
| B2 | 103.7±0.8 | 0.19±0.02 | 4.85±0.67 | 70.3 |
| B3 | 205.6±5.8 | 0.25±0.02 | 8.54±1.08 | 75.3 |
| B4 | 206.2±9.6 | 0.22±0.04 | 12.33±0.90 | 79.9 |

**Supplementary Table 7 PK parameters of VLK and IgG4 Fc fusion proteins (calculated using NCA)**

|  | **VLK-IWAT** | | **VLK-SERUM** | | **IgG4-IWAT** | | **IgG4-SERUM** | |
| --- | --- | --- | --- | --- | --- | --- | --- | --- |
|  | **AUC_0-t_** | **C_max_** | **AUC_0-t_** | **C_max_** | **AUC_0-t_** | **C_max_** | **AUC_0-t_** | **C_max_** |
| 1 | 1177.49 | 404.50 | 1851.13 | 484.13 | 2311.64 | 491.16 | 3231.74 | 722.74 |
| 2 | 738.81 | 477.00 | 1491.13 | 343.00 | 1778.78 | 590.15 | 3219.18 | 616.07 |
| 3 | 858.98 | 292.78 | 1486.90 | 311.72 | 1831.61 | 642.43 | 2823.19 | 746.51 |
| Mean | 925.09 | 391.42 | 1609.72 | 379.62 | 1974.01 | 574.58 | 3091.37 | 695.11 |
| SD | 185.09 | 75.77 | 170.71 | 75.00 | 239.71 | 62.73 | 189.70 | 56.73 |

**Supplementary Table 8 List of mice primers used in qPCR**

| **Target** | **Forward** | **Reverse** |
| --- | --- | --- |
| *B2m* | TGGTCTTTCTGGTGCTTGTCT | GCAGTTCAGTATGTTCGGCTT |
| *Fasn* | CTTCTGGTGTGCAACTGTGC | GCACTAGCAGGAAACCACCT |
| *Dio2* | GCTGACCTCAGAAGGGCT | AGGTGGTCAGGTGGCTGA |
| *Ucp1* | CTGCCAGGACAGTACCCAAG | TCAGCTGTTCAAAGCACACA |
| *Glut1* | AAAGAAGAGGGTCGGCAGAT | ACAGCGACACCACAGTGAAG |
| *Ppar-γ* | GATCCTCCTGTTGACCCAGA | TCAAAGGAATGGGAGTGGTC |
| *Fgf21* | CTGCTGGGGGTCTACCAAG | CTGCGCCTACCACTGTTCC |
| *Leptin* | GACACCAAAACCCTCAT | CAGTGTCTGGTCCATCT |
| *Scd1* | CACCTGCCTCTTCGGGATTT | AGCCGTGCCTTGTAAGTTCT |
| *Pgc1α* | ACAAAGACGTCCCTGCTCAG | TGTCTCTGCTGCTGTTCCTG |

**References**

[1] D. Bi, C. Wilhelmy, D. Unthan, I. S. Keil, B. Zhao, B. Kolb, R.I. Koning, M. A. Graewert, B. Wouters, R. Zwier, J. Bussmann, T. Hankemeier, M. Diken, H. Haas, P. Langguth, M. Barz, H. Zhang, On the Influence of Fabrication Methods and Materials for mRNA‐LNP Production: From Size and Morphology to Internal Structure and mRNA Delivery Performance In Vitro and In Vivo, Advanced Healthcare Materials, **2024**, *13*, e2401252.

[2] L. Xue, G. Zhao, N. Gong, X. Han, S. J. Shepherd, X. Xiong, Z. Xiao, R. Palanki, J. Xu, K.L. Swingle, C. C. Warzecha, R. El-Mayta, V. Chowdhary, I. C. Yoon, J. Xu, J. Cui, Y. Shi, M. G. Alameh, K. Wang, L. Wang, D. J. Pochan, D. Weissman, A. E. Vaughan, J. M. Wilson, M. J. Mitchell, Combinatorial design of siloxane-incorporated lipid nanoparticles augments intracellular processing for tissue-specific mRNA therapeutic delivery, Nature Nanotechnology **2025**, *20*, 132.

[3] A. Chan, R. M. Haley, M. A. Najar, D. Gonzalez-Martinez, L. J. Bugaj, G. M. Burslem, M. J. Mitchell, A. Tsourkas, Lipid-mediated intracellular delivery of recombinant bioPROTACs for the rapid degradation of undruggable proteins, Nature Communications **2024**, *15*, 5808.
